# Partisan differences in health behaviors can impact respiratory disease dynamics

**DOI:** 10.64898/2026.01.14.26344076

**Published:** 2026-01-19

**Authors:** Chris Soria, Audrey M. Dorélien, Dennis M. Feehan, Ayesha S. Mahmud

**Affiliations:** 1 Department of Demography, University of California Berkeley, Berkeley, CA, USA; 2 Department of Sociology, University of Washington, Seattle, WA, USA

**Keywords:** political party affiliation, health behavior, social contacts, respiratory infectious diseases, SIR model

## Abstract

The transmission of respiratory pathogens is fundamentally shaped by human behaviors such as interpersonal contacts, use of face masks, and vaccination. Political party affiliation has been shown to be associated with health-related behaviors. Yet, partisan heterogeneity in health-related behaviors is typically not included in infectious disease transmission models. Here, we leveraged uniquely detailed data from the Berkeley Interpersonal Contacts Study (BICS) on partisan differences in contact rates, mask usage, and vaccination patterns during the first year of the COVID-19 pandemic. We find substantial differences in health-related behaviors by political affiliation. Republicans reported a significantly greater number of average daily contacts, lower propensity of using masks and of getting vaccinated for COVID-19. These findings hold even after controlling for observable demographic and location-based differences across survey respondents. We adapt the classic Susceptible-Infected-Recovered (SIR) model to incorporate partisan-specific behaviors and varying levels of political homophily to simulate an outbreak of a hypothetical respiratory pathogen. We find that the observed behavior differences lead to simulated Republicans experiencing higher infection and mortality rates and earlier peaks compared to Democrats. Incorporating greater within-group mixing further amplified partisan differences in disease outcomes. Finally, we show that failure to incorporate partisan behavioral heterogeneity in disease models can lead to inaccurate predictions about the size and timing of outbreaks in a population.

## 1 Main

The dynamics of directly-transmitted respiratory diseases depend on human behaviors such as the daily number of interpersonal contacts and face mask use. Previous work has uncovered substantial demographic heterogeneities in these health-related behaviors (Adimora and Schoenbach, 2005; Okten, Gollwitzer and Oettingen, 2020); for example, age-specific differences in contact rates are a key driver of respiratory disease dynamics and age is therefore routinely incorporated in disease models (Schenzle, 1984; Ferguson, Nokes and Anderson, 1996; Dorélien et al., 2023; Feehan and Mahmud, 2021).

Partisan differences in mask-wearing, physical distancing, and vaccination emerged as salient features of the COVID-19 pandemic (Fan, Orhun and Turjeman, 2023; Gadarian, Goodman and Pepinsky, 2021, 2024), with differences often rivaling or exceeding those observed across age groups (Gollwitzer et al., 2020). These pandemic-era gaps continued a long-term trend of widening partisan differences in trust toward science and public health institutions (Blank and Shaw, 2015; Furnas, LaPira and Wang, 2025; Camacho-García et al., 2025) and in differential adherence to public health recommendations (de Hoog and Pat-El, 2024; Fraser et al., 2022). The persistence and breadth of these partisan divides suggest that partisan behavioral heterogeneity will likely persist in future disease outbreaks (Iyengar and Krupenkin, 2018; Heltzel and Laurin, 2020). But the epidemiological consequences of partisanship remain largely unquantified and partisanship is not typically included in disease models (Pacheco et al., 2024; Bedson et al., 2021).

Here, we measure partisan differences in important health related behaviors and demonstrate the importance of incorporating partisan heterogeneities in the analysis of infectious disease dynamics. We make three main contributions. First, we leverage uniquely detailed survey data on contact rates, partisanship, and behavior collected during the first year of the COVID-19 pandemic to characterize partisan differences in health-related behaviors and to identify their correlates. Second, we incorporate these empirically estimated partisan differences into widely used mathematical transmission models that simulate the spread of respiratory infectious diseases in a population. Third, we use these simulations to demonstrate the impact of partisan behavioral differences on both aggregate epidemic dynamics and partisan-specific health outcomes and disparities, following the introduction of a hypothetical novel pathogen.

We find that partisan differences in contact rates and mask usage are nearly as large as age-based differences and larger than racial and gender differences. On average, Republicans in our survey have higher contact rates, lower rates of mask usage, and lower rates of vaccination compared to Democrats. These disparities do not appear to be driven by place-based factors such as local mask mandates. Simulation results show that behavioral differences alone lead to Republicans experiencing higher infection and mortality rates compared to Democrats, as well as earlier peaks in disease incidence. These disparities are further amplified by increasing levels of within-group mixing and having a higher proportion of Republicans in the population. Lastly, we find that ignoring partisan differences in health behavior leads to inaccurate predictions of the timing and magnitude of disease peaks in the broader population. That is, models that exclude partisan heterogeneity in health behavior produce markedly different epidemic projections compared to models that incorporate it—even when both are based on the same population-average estimates of health behaviors.

## 2 Results

### 2.1 Are partisan differences in health-related behaviors large?

To quantify partisan differences in health-related behaviors, we analyzed data from the Berkeley Interpersonal Contacts Study (BICS). BICS collected information on demographic and socioeconomic characteristics, contact rates, mask usage, and vaccination rates during the first 13 months of the COVID-19 pandemic in the United States. Estimates from BICS data revealed differences in contact rates, mask usage, and COVID-19 vaccination by political party (Table 1 and Table S1). Republicans reported an average of 6.6 daily contacts, while Independents reported 5.7 contacts and Democrats reported 5.3 contacts. Among those reporting contacts, 54.8% of Republicans wore masks, compared to 53.8% of Independents and 65.6% of Democrats. In Wave 6 (May 2021), self-reported vaccination rates were 57.1% for Republicans, 60.7% for Independents, and 74.0% for Democrats. These aggregate differences also varied over time during the pandemic (Table S2), with Republicans showing the highest volatility in contact patterns (SI Temporal Patterns), a pattern consistent with state-level analyses of partisan behavior change (Urmi et al., 2025).

**Figure 1:**
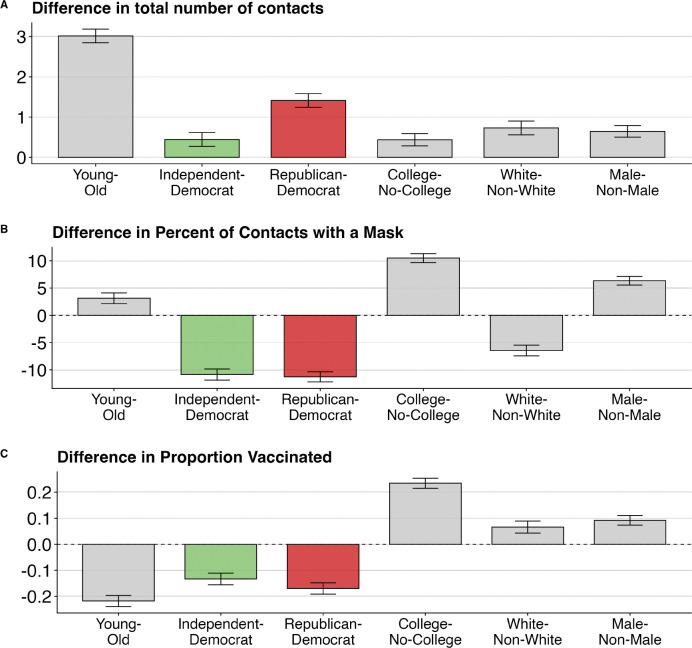
Comparison of partisan and demographic group differences for three health behaviors: (A) total reported in-person contacts from the previous day; (B) percentage of contacts involving mask use among those reporting previous-day contacts, based on detailed information from three selected contacts per respondent; and (C) estimated vaccination rates reported in Wave 6 of BICS in May 2021. Effect sizes are coefficients from univariate linear regression models estimated separately for each demographic characteristic and behavior. Sample includes respondents across six BICS survey waves (April 2020 – May 2021), except Panel C. Error bars show 95% confidence intervals.

Figure 1 illustrates the magnitude of these partisan differences in three health behaviors by comparing them with differences in the same behaviors across age, gender, race, and education groups. Republicans reported about 1.4 more average daily contacts than Democrats— a difference more substantial than the gap in average contact rates between genders, educational groups, or racial groups (Figure 1). Among those reporting contacts, Republicans and Independents were both 17% less likely than Democrats to report wearing masks (Figure 1). This partisan gap exceeded differences based on gender, race, and age. When restricting the analysis to respondents aged 65 and older, both the overall pattern and size of partisan differences remained very similar to the full sample (Fig. S2), indicating that age composition does not account for the observed partisan behavioral gaps.

Sociodemographic characteristics of BICS respondents differed across political parties: for example, Republicans in BICS were older, more likely to be white, and more likely to be male than Democrats (Table S3). These differences are consistent with other estimates of party composition (Zingher, 2018; Gadarian, Goodman and Pepinsky, 2021; Fan, Orhun and Turjeman, 2023). We use linear regression models to test whether partisan differences in each health behavior – contacts, masking, and vaccination – persist after controlling for other observed differences across individuals. In these regression models, we controlled for sociodemographic characteristics (race, gender, age group, ethnicity, education, employment, household size) and geographic-context covariates (county Democratic vote share, mortality rates, mask mandates, and state fixed effects).

**Figure 2:**
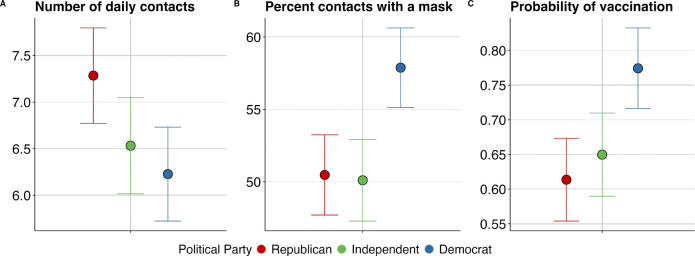
Adjusted effect sizes from multivariate linear regression models for (A) daily in-person contacts, (B) mask use, and (C) vaccination rates as of May 2021, controlling for survey design, demographics, and geographic context. Sample includes respondents across six BICS survey waves (April 2020 – May 2021), except Panel C which only includes respondents from Wave 6. Error bars represent 95% confidence intervals. The estimated marginal means are calculated for a reference individual who is a non-Hispanic White female aged 18–30, employed, college-educated, living in a metropolitan area in California during Wave 2 on a weekday, in a county with a strict mask mandate, with household size, log previous week mortality rate, and congressional district percent Democrat all set to their weighted sample averages.

Substantively large and statistically significant partisan differences persisted across all three health behaviors after controlling for individual and county-level characteristics, although estimated partisan differences were slightly attenuated (Figure 2). For example, after adding demographic, geographic, and policy controls, Republicans were predicted to have an average of 1.1 more contacts than Democrats (Table S4); Republicans exhibited 7.5 percentage-points lower mask use than Democrats (Table S5); and Republicans were roughly 16 percentage points less likely to be vaccinated than Democrats (Table S6).

Democrats also differed significantly from Independents in mask use and vaccination rates after adding controls, though these gaps were smaller than Republican-Democrat differences. These results indicate that the observed demographic and geographic differences between partisan groups do not fully explain partisan differences in health behaviors. This suggests that common epidemiological modeling approaches that stratify populations by age or other demographic characteristics would miss the independent behavioral effects of political affiliation.

### 2.2 Are partisan health-behaviors large enough to produce meaningful disease disparities?

To investigate the implications of these partisan differences for disease dynamics, we incorporated political heterogeneity in contact rates and protective behaviors into a mechanistic model for the transmission of a hypothetical respiratory pathogen. We adapted the classic Susceptible-Infected-Recovered (SIR) framework by subdividing the population into three distinct partisan groups, each with its own contact rate. To model the impact of mask-wearing, each partisan group is further divided into “protected” (P) and “unprotected” (U) subgroups (see Methods; Figure 6).

In our first set of results, we assume that the three political parties are equal in population size. We also assume that people mix randomly, i.e., without paying attention to political affiliation (this assumption is relaxed below). Remaining model parameters are chosen based on established values from the epidemiological literature (Table S7).

We evaluate three key outcomes of the mechanistic model: (1) cumulative incidence, which serves as a measure of total disease burden; (2) peak incidence, which summarizes the intensity of the outbreak; and (3) time to peak incidence, which serves as a measure of how rapidly the disease progresses through the population.

**Figure 3:**
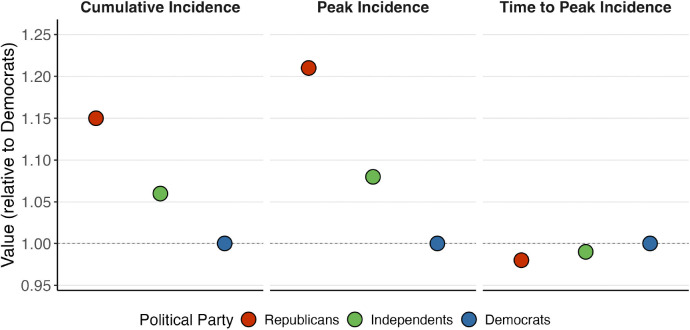
Simulation results from a three-party SIR model for (A) cumulative incidence (B) peak infection as a percentage of the population, and (C) time to peak infection. Results are from a one-year simulation for a hypothetical respiratory pathogen, with homogeneous partisan mixing and equal partisan group sizes. Partisan contact rates and rate of adoption of protective behavior are obtained from BICS data. All other model parameters were sourced from published literature as detailed in Table S5. All outcomes are shown as values relative to Democrats (reference = 1); > 1 indicates higher than Democrats, < 1 indicates lower than Democrats.

Figure 3 summarizes simulation results by comparing key disease outcomes across partisan groups, with all metrics standardized relative to Democrats (value = 1). Using only the aggregate behavioral differences in contact rates and mask usage documented above (Section 2.1)—while holding all other factors equal—simulated Republicans experience a 15% higher cumulative incidence than Democrats after one year. Republicans also face a peak incidence about 21% higher than Democrats, with the peak arriving roughly two days earlier.

The higher transmission rate experienced by Republicans—due to higher contact rate and lower rate of adopting protective behavior—results in a quicker depletion of their susceptible pool and a larger proportion that are eventually infected and recovered, compared to Democrats and Independents (Fig. S4). In general, in our simulations, contact rates play a larger role than mask usage in driving these partisan differences in disease outcomes (SI Decomposing Effects).

Many infectious diseases, such as COVID-19 and influenza, have a strong age-related mortality gradient (Sorci, Faivre and Morand, 2020); if the age composition of political parties differ, this could lead to different case fatality rates for each political party. As a sensitivity analysis, we allow the case fatality rate to vary across partisan groups to account for differences in their age composition; this produces a higher case fatality rate for Republicans, who tend to be older. In this scenario, the mortality results change dramatically: Republicans show a 75% higher mortality rate than Democrats (Fig. S6), a difference that reflects the combined effect of their older age distribution and behavioral factors.

To gauge how much partisan behavior drives respiratory infectious disease dynamics, we also reran the simulations under a counterfactual “cautious population” scenario. In this scenario, all partisan groups adopt the population-average behavior of the partisan group with the lowest-transmission profile observed in the survey, i.e. the fewest social contacts combined with the highest mask-use rate. Fig. S5 shows that moving the entire population to this best-case behavioral scenario would reduce the epidemic peak by 47% and avert about 18% of deaths relative to the simulations with partisan heterogeneity in behavior. All groups have lower incidence and mortality, but the benefits are unequally distributed: Democrats would experience 12% fewer deaths, while Republican deaths would drop by 25% and their peak caseload would fall by 51% (Table S8). In short, if every partisan group adopted the behaviors of the most cautious group, the outbreak’s overall severity would decline dramatically. A population universally adopting this lowest-transmission behavior would also markedly delay the time needed to reach the outbreak peak by nearly three months, extending the epidemic’s duration while reducing its intensity.

### 2.3 Do homophily and population composition moderate partisan disease disparities?

Having established that partisan behavioral differences alone produce meaningful disease disparities (Section 2.2), we next examined how partisan homophily and population composition moderate these outcomes. We accounted for the possibility of homophily by political affiliation in our model by including a parameter, β, that controls the extent to which contacts are disproportionately likely to happen within partisan groups. In the random mixing scenario discussed above, β=1; when β>1, contacts are more likely within partisan groups. We accounted for partisan composition in our model by including two parameters that govern the share of the population that is Republican and Democrat. We repeated the simulation described above for all combinations of β∈[1,5] and share of Republicans and Democrats between 20 and 70%.

**Figure 4:**
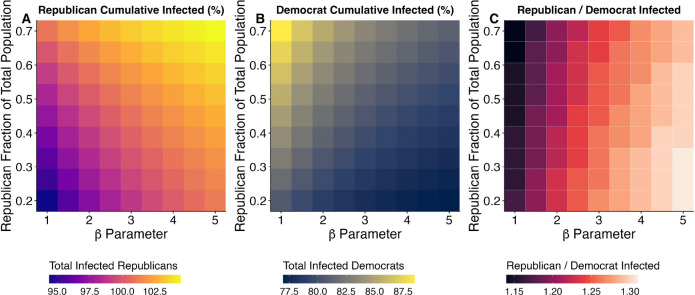
Simulated cumulative infections for (A) Republicans and (B) Democrats, and (C) the ratio of the number of Republican infections to Democrat infections. Results are from simulations with varying Republican homophily parameters (β: 1.0–5.0) and population fractions using the three-party SIR model with base parameters from Table S7. X-axis shows increasing partisan homophily (higher values = stronger in-group contact probability); y-axis shows Republican population proportion (20–70%) with Democrats comprising the remainder and Independents fixed at 10%.

Figure 4 shows how homophily and population composition interact to influence cumulative infections after one year among Republicans (panel A) and Democrats (panel B). For instance, in Figure 4 Panel A, coordinates (1, 0.2) show that when β=1 (random mixing) and when Republicans constitute 20% of the population, (and Democrats 70%, Independents 10%), about 95% of Republicans end up infected after a year. Conversely, at the opposite extreme where homophily is high (e.g., β=5) and Republicans make up 70% of the population, the cumulative infections exceed 100% among Republicans (this includes repeat infections). Figure 4 reveals that as homophily increases (β increases along the x-axis) and a greater share of contacts occur within partisan groups, cumulative infections for Repbulicans increase and cumulative infections for Democrats decrease. As the Republican fraction of the population increases (increasing values along the y-axis), a greater number of contacts for all parties occurs with Republicans, increasing cumulative infections for both Republicans and Democrats (Fig. S7 shows the underlying partisan-specific epidemic curves for selected scenarios).

Fig. S8 shows parallel results for the other disease outcomes. Republican mortality, peak incidence, and timing of peak infections become more severe with stronger partisan homophily and larger Republican population shares; both factors increase within-group contacts among Republicans (Fig. S11), who have higher contact rates and lower rates of adpoting protective behaviors. This increases the force of infection for Republicans, thereby increasing disease burden (Figure 4A; Fig. S8A,C,E). In scenarios combining high homophily and a large Republican majority, cumulative incidence surpasses 100% (Table S9), with epidemic peaks occurring up to 46 days earlier compared to Democrat-majority populations under random mixing. However, when Republicans represent 70% of the population, increasing homophily from β=1 to β=5 advances the Republican peak by only 5 days, indicating reduced sensitivity of peak timing to homophily under Republican-majority conditions (Fig. S8E–F).

Democrat cumulative incidence, on the other hand, decreases as both homophily and the proportion of Democrats in the population increase (Figure 4B). Higher homophily benefits Democrats by increasing within-group contacts and reducing their contacts with Republicans ((Fig. S12), thereby lowering their force of infection. Higher homophily also increases disparities between Democrats and Republicans’ cumulative incidence rates; the highest disparities occur when share of Democrats are high (see Figure 4C). Even in contexts where Democrats are a small minority within Republican-majority populations, increasing homophily consistently reduces Democrat cumulative incidence. However, as the Republican share of the population rises, Democrats experience increased cumulative incidence due to greater number of contacts with Republicans. Across all simulations with varying homophily and population composition, Democrat cumulative incidence rates (Fig. S9 B,D) consistently remain below Republican incidence rates (Fig. S9, A,C).

In the previous section, we show that when there is no homophily and partisan groups are equal in size, Republican cumulative incidence is 15% higher than that of Democrats. Introducing partisan homophily and variation in population composition greatly affects this relative difference in outcomes. For example, when Republicans constitute 50% of the population and exhibit high within-group mixing (β=5), cumulative incidence is 31% higher in Republicans than in Democrats (Figure 4, Panel C).

Similarly, mortality, peak incidence, and timing of peak infections among Democrats generally decrease as homophily and the proportion of Democrats in the population increase (Figure 4B and Fig. S8B,D,F).

### 2.4 Do partisan behavioral differences impact overall epidemic projections?

The vast majority of respiratory disease transmission models used for simulations and forecasts either do not account for heterogeneities in the force of infection across population subgroups, or only do so by age.

We find large differences in contact rates and other behaviors observed across partisan groups, yet disease models do not typically incorporate political heterogeneity.

Here, we investigate whether or not ignoring partisan differences has an impact on population-level disease models in a setting where partisan differences are large. We construct a hypothetical population in which partisan health behaviors match average values observed in BICS, and assuming high levels of homophily (β=5). We call this the *homophilous partisan heterogeneity model*. Using this model as the reference, we then investigate how well the aggregate dynamics of the outbreak can be approximated using two simpler models: (1) the *partisan heterogeneity model*, which assumes that contact and mask usage rates vary by party, but that does not account for homophily; and (2) the *uniform behavior model*, which disregards partisanship completely and instead models population average behaviors observed in BICS.

To explore how population composition moderates these effects, we examine each model across three distinct population compositions: an evenly split population (R: 33%, D:33%, I: 33%), a Republican-majority population (R: 70%, D: 20%, I: 10)%, and a Democrat-majority population (R: 20%, D: 70%, I: 10%).

**Figure 5:**
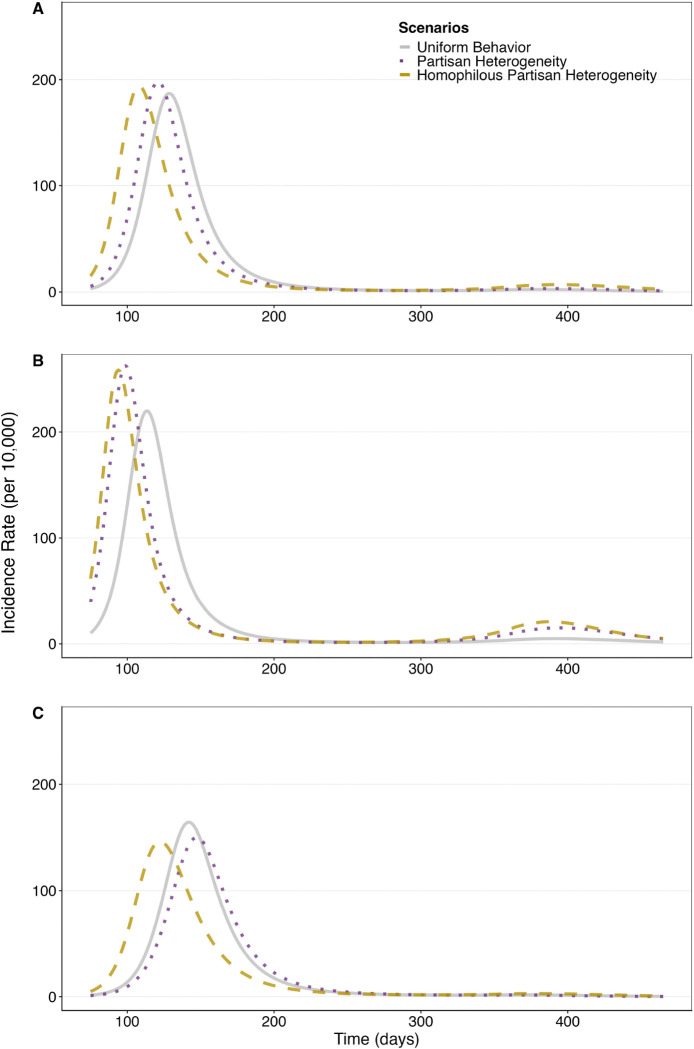
Simulated incidence rates per 10,00 in (A) an evenly split population, (B) a Republican majority population, and (C) a Democrat majority population. Gray solid line shows theuniform behavior model using population-average behaviors; purple dotted line shows the same population with partisan behavioral heterogeneity; yellow dashed line shows the partisan heterogeneity model with strong in-group preference (β=5).

Figure 5 shows the results for an evenly split population (panel A), for a heavily Republican population (panel B), and for a heavily Democratic population (panel C). Within each of these Panels A, B, and C in 5, we assume the same population-average contact rates and health behaviors for all three scenarios. In an evenly split population, the simpler models (Uniform Behavior and Partisan Heterogeneity) predict a similar cumulative incidence rate as the Homophilous Partisan Heterogeneity model but over-estimate the time it takes for the outbreak to reach its peak: the partisan heterogeneity model predicts peak incidence 8 days late, and the uniform behavior model predicts peak incidence 20 days late (panel A; Table S8, Table S10).

In a population where Republicans are the majority, both of the simpler models have a later peak compared to the Homophilous Partisan Heterogeneity scenario (Figure 5 B and Table S9). Relative to this reference, the Partisan Heterogeneity Model without homophily delays the peak by 4 days with marginally different outcomes. The Uniform Behavior Model shows the largest deviations: delaying the peak by 19 days, reducing peak prevalence from 2,100 to 1,869 per 10,000, and lowering cumulative mortality from 135 to 114 deaths per 10,000. In Republican-majority populations, homophily produces comparatively smaller changes in epidemic projections because demographic composition alone ensures high intra-group contact rates among Republicans, even under random mixing assumptions (Fig. S11, B).

In a population where Democrats are the majority, the Partisan Heterogeneity Model without homophily delays the peak by 24 days and moderately increases the final outbreak size compared to the Homophilious Partisan Heterogeneity model (Table S11). The Uniform Behavior Model shows even larger deviations, delaying the peak by 18 days from the reference while substantially increasing cumulative mortality. In this Democratic-majority composition, the minority Republican subgroup drives these epidemic dynamics, as homophily concentrates their within-group interactions (Fig. S11, C), accelerating overall transmission while reducing peak infections among the Democratic majority.

Overall, these results demonstrate that incorporating partisan heterogeneity and homophily into epidemiological models is critical for accurate outbreak projections, as simpler approaches systematically underestimate epidemic speed and, depending on population composition, may substantially misjudge disease burden.

## 3 Discussion

Our analysis produced three key insights: first, partisan disparities in health behaviors are substantial in magnitude even after controlling for demographic and geographic factors; second, incorporating these substantial partisan differences in disease models reveals corresponding disparities in outbreak timing and disease burden; and third, these partisan differences substantially alter aggregate disease spread projections, particularly when modeled with assumptions about partisan homophily.

Partisan differences in protective health behaviors are more pronounced than those associated with gender, race, or education—factors widely considered meaningful enough for inclusion in epidemiological models (Manna et al., 2024; Buckee, Noor and Sattenspiel, 2021; Tizzoni et al., 2022; Bedson et al., 2021). These partisan gaps cannot be attributed to demographic or geographic variation, both of which are commonly adjusted for when accounting for behavioral heterogeneity in disease modeling (Mistry et al., 2021).

Disease models reveal that these partisan behavioral differences translate into significant disparities in partisan-specific disease outcomes. Under random mixing contact patterns (β=1) where partisan groups are assumed equal in size, Republicans experience 15% higher incidence after one year; when combined with their older age distribution, this results in 75% higher mortality (Figure 3). When we incorporate homophilous mixing, disparities increase substantially (up to 31% higher relative incidence), highlighting the importance of incorporating partisan homophily into disease models (Figure 4); this finding is consistent with other studies that have shown that homophily can substantially alter epidemic dynamics (Smaldino and Jones, 2021; Thomas et al., 2020). Research shows that levels of partisan homophily in contact are plausibly high (Kalmoe and Mason, 2022; Iyengar and Westwood, 2015; Iyengar and Krupenkin, 2018; Brown and Enos, 2021; Huber and Malhotra, 2017) but, unfortunately, no existing contact surveys directly measure partisan homophily. Since it seems unlikely that survey respondents can reliably identify their contacts’ political affiliations, future research should prioritize developing statistical methods that estimate partisan homophily indirectly through observable network and geographic patterns.

Partisan heterogeneity also fundamentally alters population-level epidemic projections by shifting outbreak timing and magnitude, even when simple models accurately capture overall population behaviors. Models ignoring partisan heterogeneity make inaccurate predictions about critical outcomes such as time to outbreak peak, with the largest discrepancies occurring in Democrat-majority populations (Figure 5). Although a one- to two-week discrepancy may seem modest, such mistiming could critically impair hospital surge planning, resource allocation, with potentially substantial mortality impacts (Geoffrey French et al., 2021; Amuedo-Dorantes, Kaushal and Muchow, 2021)

Several limitations of our analysis point to important directions for future research. First, we do not have data on partisan differences in contact rates *prior* to the COVID-19 pandemic. Although our analysis focuses on health behavior data collected during the COVID-19 pandemic, partisan differences in health behaviors are not new; longstanding and widening partisan divides in attitudes toward science (Kennedy, 2024), health policy (Fraser et al., 2022; Gollust et al., 2024), and vaccination (Baum, 2011; Jones and McDermott, 2022) suggest these behavioral gaps will most likely persist in future epidemic and pandemic contexts (Fan, Orhun and Turjeman, 2023; Gadarian, Goodman and Pepinsky, 2021, 2024). Future social contact studies are needed to determine whether these differences persist outside of pandemic contexts. Additionally, partisan health behaviors likely vary across geographic settings (Mistry et al., 2021), and future work should examine how local context shapes these behavioral patterns. Collecting data on prevalence- and mortality-dependent behavior by political party affiliation would also be valuable; although not shown in this paper, we found preliminary evidence that Republicans may exhibit mortality-dependent behavior, which could help reduce disparities in health outcomes. Finally, our analysis focused on health outcomes, but protective behaviors carry other costs and benefits—including economic, social, and psychological dimensions—that warrant further investigation. These data limitations are not merely technical gaps. Without accurate measurement of partisan homophily and the context-dependence of behavioral divides, models used to guide resource allocation, vaccination campaigns, and intervention timing may systematically misestimate outbreak risk in politically homogeneous communities—precisely the populations where behavioral clustering is strongest. Given that future pandemics are expected (Marani et al., 2021), these modeling errors could result in preventable loss of life.

## 4 Data and Methods

### Data

The Berkeley Interpersonal Contacts Study (BICS) used surveys to collect detailed data on demographic and socioeconomic characteristics, contact rates, and health-related behaviors during the first 13 months of the COVID-19 pandemic in the United States. Respondents were asked how many people they had face-to-face contact with on the day before the survey interview, and detailed information was collected on up to three of these contacts, including information about each contact’s age, sex, race/ethnicity, mask usage, and physical distance. Repeated cross sections were recruited from an online panel and weighting methods were developed to make the sample representative of the national population. Data collection started with a pilot wave in March 2020 (Wave 0) and continued with six additional waves conducted over the next year and a half. Additional details on the survey methodology, questionnaire design, sampling, and methods used for weighting are described in (Feehan and Mahmud, 2021).

We analyze data from BICS waves 1 through 6, spanning April 2020 to May 2021. Wave 0 is omitted because it did not record political variables in a comparable way. Political party identification was assessed by the panel provider prior to the survey. Upon enrollment in the panel, all potential survey respondents were asked to select their partisan affiliation from standard categories (Democrat, Republican, Independent, or Prefer not to Say/Other). We exclude respondents in the “Prefer not to Say/Other” category, to prevent potential misclassification bias. The analytical sample therefore comprises respondents in three discrete self-identified partisan categories: Democrats, Republicans, and Independents.

We focus on three measures of health behavior. First, we examine average daily contacts, measured through the question “How many people did you have in-person contact with yesterday?” and through reports about the respondent’s household members. Second, for respondents reporting non-zero contacts outside the household, we assessed mask-wearing behavior for up to three contacts, measured through the question “During this contact, did you wear a face mask?” For this measure we calculate the percent of contacts with a mask. Third, we analyze self-reported COVID-19 vaccination status (“Have you gotten vaccinated against COVID-19?”) from Wave 6 (May 2021) data, when vaccines had become accessible to the general public. Our measure of interest is percent vaccinated.

### Statistical model to characterize partisan differences

We first investigate the relationship between partisan identity and health behaviors using regression models. We pool the data across survey waves and, for each of the three measures of health behavior (described above), we estimate the following regression model:

(1) $$y_{i}=\beta_{0}+\beta_{R}R_{i}+\beta_{I}I_{i}+S_{i}^{⊤}\beta_{S}+D_{i}^{⊤}\beta_{D}+C_{i}^{⊤}\beta_{C}+\epsilon_{i}.$$

where $y_{i}$ is the health-behavior measure for respondent $i;\;R_{i}$ and $I_{i}$ are indicators for Republican and Independent identification, respectively, with Democrats as the omitted category; $S_{i}$ is a vector of survey characteristics (wave, day, city); $D_{i}$ is a vector of sociodemographic characteristics for i (race, gender, age group, ethnicity, education, employment, household size); and $C_{i}$ is a vector of geographic-context covariates (county Democratic vote share, mortality rates, mask mandates, and state fixed effects). The model uses survey weights to account for the study design.

The coefficients on the binary indicators for Republican ($\beta_{R}$) and Independent ($\beta_{I}$) represent the adjusted differences in health behaviors relative to Democrats, controlling for sociodemographic composition (see Table S3), survey characteristics, and geographic area.

We also estimated models more suited to the data structure (negative binomial for contact counts, logistic regression for vaccination outcomes), but results were substantively identical. We present linear models for ease of interpretation.

### Disease transmission model

**Figure 6:**
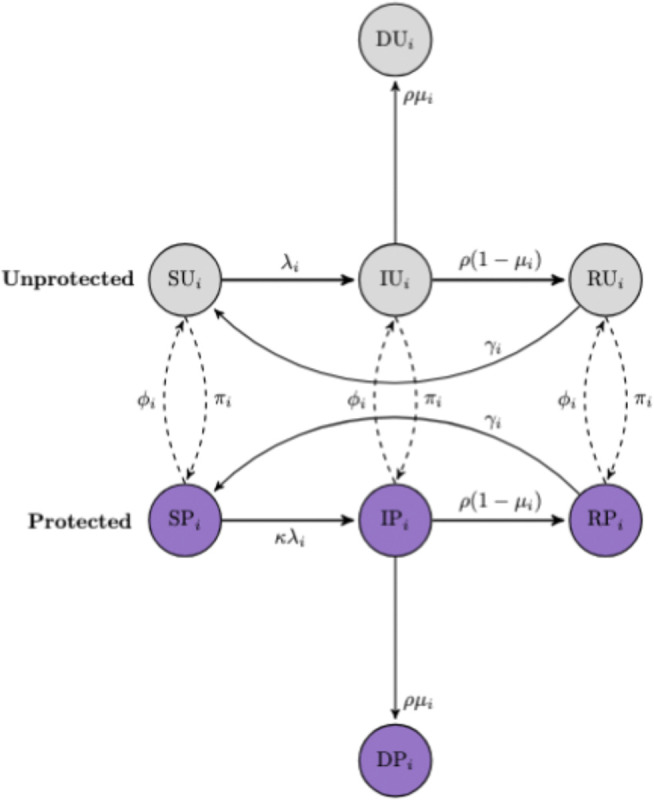
Flow diagram showing movement between unprotected and protected SIR classes

We next investigate the implications of partisan differences in behavior for the dynamics of a hypothetical respiratory disease outbreak. We adapt an SIR model of respiratory disease transmission to capture heterogeneities in health-related behavior and interactions across the three partisan groups. In the SIR compartmental model, the population is divided into three mutually exclusive groups: susceptible, infected, and recovered. We also subdivide the population into three distinct partisan groups. To model the impact of mask-wearing, each partisan group is further divided into “protected” (P) and “unprotected” (U) subgroups (Harris, Cardenas and Mordecai, 2023; Smaldino and Jones, 2021) (Figure 6). Individuals in the protected subgroups have a lower probability than individuals in the unprotected subgroups of acquiring the infection following contact with an infected individual, as well as a lower probability of transmitting the infection to a susceptible individual. Unprotected individuals in political party i adopt protective behaviors at rate $\pi_{i}$ and move to the P subgroup; to capture fatigue and waning of protective health behaviors, we model a low rate of movement, $\phi_{i}$, back from protected to unprotected status (Figure 6).

Regardless of whether the individual is in the U or P subgroups, the flow between the SIR compartments follows the typical disease transmission process, where susceptible individuals acquire infection through contact with an infected individual, and recover after a certain time period. The force of infection, $\lambda_{i}$, for susceptible individuals in political party, i, is given by:

(2) $$\lambda_{i}=\tau \left(\sum_{j=1}^{3}\;{\overset{‾}{c}}_{ij}\left(\frac{IU_{j}}{N_{j}}+\kappa \frac{IP_{j}}{N_{j}}\right)\right)$$

Where:

- τ is the baseline transmission probability per effective contact;
- ${\overset{‾}{c}}_{ij}$ is the average number of contacts per unit time that one individual in party i has with people in party j;
- $\frac{IU_{j}}{N_{j}}$ represents the proportion of party j in the infected-unprotected class;
- $\frac{IP_{j}}{N_{j}}$ represents the proportion of party j in the infected-protected class;
- and κ is the protection scaling factor that reduces the probability of transmission for contacts that involve protected individuals.

Individuals in the infected compartment either recover (at rate ρ) or die (at rate μ). Over time, immunity wanes at rate γ, moving individuals from the recovered compartment back to the susceptible compartment.

To ensure comparability across groups, at the start of the simulation, we seed the infection by introducing one infected individual into each partisan group. We assume the population is fully susceptible to the pathogen at the start of the simulation, and run the simulation for one year. For all simulations, we assume the total population size is 10 million. We investigate several versions of the model to unpack the effects of partisan differences in health behaviors; the population composition of partisanship; patterns of homophily by partisanship in the contact network; and partisan responsiveness to epidemic conditions. Although we highlight differences in vaccination rates between partisan groups in Wave 6 of the survey, we do not mechanistically model the impact of vaccination, as we only had a single wave of data on vaccination at the start of the COVID-19 vaccination campaign, when the vast majority of people were still ineligible for the vaccine. These simulations are intended to provide insight into how partisan differences might affect the dynamics of a typical respiratory disease; they are not intended to exactly model COVID-19 or any other specific historical outbreak.

### Components of the SIR model that vary by party

Patterns of contact rates and mask use for each partisan group were based on group averages across all waves in the BICS data (Table 1). Because we are interested in population-level simulations rather than time or location-specific modeling, we used survey data weighted to be nationally representative and averaged across all six waves of the survey to calculate the average contact rates by party affiliation.

We chose values for the adoption of protective behavior ($\pi_{i}$) and the waning protection rate ($\phi_{i}$) to match the general pattern of party-specific mask-wearing rates observed in the BICS data over time (Table S7).

### Homophily and Relative Size of Partisan Group

We model two additional factors that can play an important role in the structure of contacts: homophily and partisan composition. Previous research has revealed that contact networks often exhibit moderate to high levels of homophily: people who are similar to each other are more likely to have contact with one another (McPherson, Smith-Lovin and Cook, 2001). We account for the possibility of homophily by political affiliation in our model by including a parameter β that controls the extent to which contacts are disproportionately likely to happen within partisan groups (Currarini, Jackson and Pin, 2010). The relationship between β and average daily contact rates between and within partisan groups is described in detail in Supplementary Section 0.1. Briefly, when β=1, contacts are independent of partisan affiliation. As β increases above 1, homophily increases and people interact more with others who have the same political affiliation relative to those with a different affiliation. We cannot directly measure partisan homophily from the BICS data, so we systematically vary the homophily parameter (β∈[1,5]) and investigate how changing homophily affects the dynamic of an outbreak.

Partisan composition also affects contact structure; for example, larger Republican populations increase the likelihood of all individuals having more contacts with Republicans. We account for partisan composition in our model by including parameters that governs the share of the initial population that are Republican and Democrat. There is no typical value to use for this parameter, since partisan composition varies considerably across the United States. For example, in 2024, the two-party share of people who are registered as Republicans varied from a low of 8% in Massachusetts to a high of 80% in Wyoming (NCSL, 2025). We therefore also explore a range of values of partisan composition (Republican share 20%-70%).

## Supplementary Material

Supplement 1

## Figures and Tables

**Table 1:** Pandemic Behaviors by Political Affiliation

Reported Behavior	Republicans	Independents	Democrats

Total Daily Contacts	6.6	5.7	5.3
Non-Household Contacts	4.7	3.9	3.5
% Contacts with a Mask	54.8	53.8	65.6
% Vaccinated	57.1	60.7	74.0

N	5,517	4,527	8,189

Descriptive statistics from pooled BICS survey data (April 2020–May 2021) weighted for national representativeness. Contact behaviors measured as previous-day reports; mask use calculated among respondents reporting contacts; vaccination rates as of wave 6 (May 2021).

## Data Availability

All study data are available at https://dataverse.harvard.edu/dataset.xhtml?persistentId=doi:10.7910/DVN/M74AJ4 All code for replicating the analysis are available at https://github.com/chrissoria/bics-partisan-disease-paper. A Shiny App for exploring model simulations is available at https://huggingface.co/spaces/bics-berkeley/partisan-disease-simulator.

## Significance

The timing and size of infectious disease outbreaks are shaped by health-related behaviors that affect disease transmission. Using data specifically designed to measure interpersonal contacts and other health behaviors, we find that during the COVID-19 pandemic, Republicans reported a significantly greater number of average daily contacts, lower propensity of using masks and of getting vaccinated. These observed differences lead to Republicans experiencing higher infection and mortality rates and earlier peaks compared to Democrats in a model simulating a hypothetical respiratory infection. Incorporating a preference for within-group mixing further amplified partisan differences in disease outcomes. Failure to incorporate partisan behavioral heterogeneity in disease models can lead to inaccurate predictions about the size and timing of outbreaks in a population.
