# Supplementary material for: Partisan differences in health behaviors can impact respiratory disease dynamics
Source: medRxiv. 2026 Jan 19:2026.01.14.26344076. Preprint. [Version 1] doi: 10.64898/2026.01.14.26344076 (PMC12879710; doi:10.64898/2026.01.14.26344076)
Supplement: Supplement 1 [file NIHPP2026.01.14.26344076v1-supplement-1.pdf]

medRxiv preprint doi: https://doi.org/10.64898/2026.01.14.26344076; this version posted January 19, 2026. The copyright holder for this preprint (which was not certified by peer review) is the author/funder, who has granted medRxiv a license to display the preprint in perpetuity.
It is made available under a CC-BY 4.0 International license .

Supporting Information: Partisan Differences in Health Behaviors and Disease Outcomes

medRxiv preprint doi: https://doi.org/10.64898/2026.01.14.26344076; this version posted January 19, 2026. The copyright holder for this preprint (which was not certified by peer review) is the author/funder, who has granted medRxiv a license to display the preprint in perpetuity.
It is made available under a CC-BY 4.0 International license .

Supporting Information Appendix

0.1 Key Term Definitions

Contacts: We define contacts as in-person physical or conversational interactions with other individuals. Conversational contacts were defined as (two-way conversation with three or more words in the physical presence of another person. This encompasses a wide range of encounters, from brief exchanges with cashiers to extended interactions with family members or fellow churchgoers.

Homophily: The homophily parameter β determines the degree of within-group interaction. When this parameter is set to 1, it implies random mixing based purely on the relative sizes of the groups. Values greater than 1 indicate a preference for in-group contacts, while values less than 1 would indicate a preference for out-group contacts (Figure S10).

Mask Use (Percent of Contacts with a Mask): For respondents reporting non-zero contacts outside the household, we assessed mask-wearing behavior for up to three contacts using the question "During this contact, did you wear a face mask?" We calculate the percentage of these reported contacts during which the respondent wore a mask.

Protected/Unprotected Subgroups: Within each partisan group in our SIR model, individuals are further classified as either "protected" (P) or "unprotected" (U) based on their adoption of protective health behaviors such as mask-wearing. Individuals in protected subgroups have a lower probability of acquiring infection following contact with an infected individual, as well as a lower probability of transmitting infection to susceptible individuals.

Cumulative Incidence: The total proportion of the population that has been infected over the course of the simulation. This measure captures the overall disease burden experienced by a group or population.

Peak Incidence: The maximum number of new infections occurring per unit time during the outbreak. This measure summarizes the intensity of the epidemic at its most severe point and is relevant for understanding healthcare system strain.

Uniform Behavior Model: A simulation scenario that treats the population as homogenous, with average contact rates and mask usage values set at values observed in BICS data aggregated across all partisan groups.

Partisan Heterogeneity Model: A simulation scenario that incorporates partisan-specific contact rates and mask usage behaviors but assumes random mixing across partisan groups (i.e., no homophily, $\beta = 1$).

Homophilous Partisan Heterogeneity Model: A simulation scenario that incorporates both partisan-specific health behaviors and preferential within-group mixing ($\beta = 5$), representing a population where individuals are more likely to have contact with others of the same political affiliation.

medRxiv preprint doi: https://doi.org/10.64898/2026.01.14.26344076; this version posted January 19, 2026. The copyright holder for this preprint (which was not certified by peer review) is the author/funder, who has granted medRxiv a license to display the preprint in perpetuity.
It is made available under a CC-BY 4.0 International license .

0.2 Decomposing the effects of specific health behaviors and comparing partisan differences in outcomes

Although both mask use and contact patterns influence the severity of the Republican epidemic curve—resulting in higher peak prevalence, earlier peak timing, and greater cumulative mortality—contact rates play a more substantial role. Differences in mask usage alone result in 5.4% more Republican deaths relative to Democrats after one year, whereas differences in contact rates lead to a 10.4% gap (Table S8).

Incorporating both differences in mask usage and contact rates into the "Partisan Heterogeneity" model amplifies the difference in outcomes between Republicans and Democrats. Republicans exhibit approximately 16% higher death rates than Democrats after one year, growing to 17% after two years (Table S8). When accounting for the older average age of Republicans, the cumulative death disparity increases dramatically to over 75% after one year (Figure S6).

Interestingly, although Democrat behaviors are most protective in the Masks + Contacts model, peak prevalence is highest here for them (15.7%) and the time needed to reach that peak is shortest (130 days). Simultaneously, the percent Democrat deceased is smallest in this model. This occurs because, as we incorporate more Republican behaviors, which tend to be less cautious, a higher proportion of Democrat infections come from Republicans in this scenario where we do not incorporate any homophily. Given Republicans' less cautious behavior, their group's peak comes sooner (128 days) and thus they reach outbreak peak sooner (Table S8).

medRxiv preprint doi: https://doi.org/10.64898/2026.01.14.26344076; this version posted January 19, 2026. The copyright holder for this preprint (which was not certified by peer review) is the author/funder, who has granted medRxiv a license to display the preprint in perpetuity.
It is made available under a CC-BY 4.0 International license .

0.3 Temporal patterns in partisan health behaviors

Beyond these aggregate differences, temporal analysis reveals important patterns in how these behaviors evolved over the first year and half of the COVID-19 pandemic. Analysis of data across survey waves revealed that partisan contact rates and masking rates fluctuated considerably over time (Figure S1). In every survey wave, Democrats exhibited greater protective health behaviors compared to Republicans. Over time, Democrats monotonically increased their contact rates, while there was more volatility in contact rates of Republicans and Independents (Figure S3). Quantitative analysis confirmed this pattern, with Republicans showing the highest relative volatility in contact patterns (coefficient of variation = 0.26) compared to Independents (0.19) and Democrats (0.16). These differences in variance were statistically significant (Levene's test: $F = 15.47$, $df = 2$, $p < 0.001$), indicating group-level variability across political parties (Table S12).

medRxiv preprint doi: https://doi.org/10.64898/2026.01.14.26344076; this version posted January 19, 2026. The copyright holder for this preprint (which was not certified by peer review) is the author/funder, who has granted medRxiv a license to display the preprint in perpetuity.
It is made available under a CC-BY 4.0 International license .

0.4 Disease Model Parameters and Equations

We develop a susceptible-infected-recovered (SIR) model that incorporates partisan differences in contact behavior and in adoption of protective behavior. The population is divided into three partisan groups, corresponding to Democrats, Republicans, and Independents; we index these groups using i. Within each partisan group i, there are four disease statuses: Susceptible, Infected, Recovered, and Deceased; and there are also two protective behavior statuses: Unprotected and Protected. All together, each partisan group has eight compartments:

- SU_i, SP_i: Susceptible individuals (unprotected, protected)
- IU_i, IP_i: Infected individuals (unprotected, protected)
- RU_i, RP_i: Recovered individuals (unprotected, protected)
- DU_i, DP_i: Deceased individuals (unprotected, protected at time of death)

At any point in time, the total population in group i is:

$$N_i = \mathrm{SU}_i + \mathrm{SP}_i + \mathrm{IU}_i + \mathrm{IP}_i + \mathrm{RU}_i + \mathrm{RP}_i. \tag{1}$$

Note that deceased people are removed from the population.

Key Features of Model Dynamics

Model dynamics are specified as a system of ordinary differential equations; these are written out in detail in a section below and also depicted in Figure 6. Model parameters are also summarized in Table **??**.

Here, we discuss two key features of our model in more detail: first, we explain how contacts work; second, we explain how contacts produce infections.

Contacts. In our model, contacts are a function of partisan group. The contact matrix $\mathbf{C}$ contains entries $\bar{c}_{i,j}$, which have the average number of daily contacts that a single person in group i has with people in group j. We also call $\bar{c}_i = \sum_j \bar{c}_{i,j}$ the average number of contacts that a single member of group i has with people in all groups.

Cross-group contacts must satisfy a balance constraint: the total number of contacts from group i to group j must equal those from group j to group i:

$$N_i \, \bar{c}_{ij} = N_j \, \bar{c}_{ji}, \tag{2}$$

where N_i, N_j are the total number of people in group i and j.

Homophily: Modeling preferential in-group contact. Homophily refers to a near-universal pattern found in human social networks: people are disproportionately likely to be connected to others who are similar to themselves (McPherson, Smith-Lovin and Cook, 2001; Currarini, Jackson and Pin, 2010). Previous analyses of contact networks have generally found strong evidence of homophily by age (e.g., Mossong et al. (2008)). Relatively few studies have investigated mixing in contact networks beyond age, but a few studies have found evidence of evidence of homophily by additional characteristics, including race/ethnicity and social groups such as class, sports team, work group, etc. (e.g., Pasquale et al. (2024); Glass and Glass (2008); Huang et al. (2016); Potter, Smieszek and Sailer (2015)).

medRxiv preprint doi: https://doi.org/10.64898/2026.01.14.26344076; this version posted January 19, 2026. The copyright holder for this preprint (which was not certified by peer review) is the author/funder, who has granted medRxiv a license to display the preprint in perpetuity.
It is made available under a CC-BY 4.0 International license .

We are not aware of any study that documents the extent of political homophily in interpersonal contact networks, but the widespread evidence of homophily in contact and other social networks, together with the spatial and demographic clustering of political affiliations (e.g., Brown and Enos (2021)), make it highly plausible that interpersonal contact networks are homophilous with respect to partisan identity. We therefore introduce a parameter in our disease model that controls the extent to which contacts are more likely to take place among members of the same partisan group. We adopt a parameterization of homophily inspired by Currarini, Jackson and Pin (2010).

Note that, in our model, homophily by partisan identity could arise as a result of personal preferences – but it could also result from structural factors that make it more likely for same-partisan people to encounter one another. We do not speculate about the possible origins of partisan homophily here; instead, our goal is to understand its possible consequences.

Formally, recall that N_i is the number of people in group i; let $M_i = N_i\ \bar{c}_i$ be the total number of contacts that all members of group i have per unit of time; and let $M = \sum_i M_i$ be the total number of contacts the entire population has per unit of time. We define the share of all contacts that is attributable to group i to be

$$m_i = \frac{M_i}{M} = \frac{N_i\ \bar{c}_i}{\sum_j N_j\ \bar{c}_j}. \tag{3}$$

So, if we sampled a contact at random from the population and chose one of the two people involved, the chances of that person being in group i would be m_i.

Let q_{ij} be the share of group i's contacts that take place with people in group j. Then the average number of contacts someone in group i has with people in group j, $\bar{c}_{ij} = \bar{c}_i\ q_{ij}$.

We focus on populations with three groups, but this setup could be generalized to additional groups in other contexts. We introduce a parameter $\beta \geq 1$ which controls the amount of homophily in contacts by tuning the extent to which the first group's contacts tend to be with other members of the first group and the extent to which the second group's contacts tend to be with other members of the second group. We cannot then independently vary contacts within the third group, as we shall see below.

Our approach takes several values as given, and then uses those values plus assumptions which we describe below to produce the matrix $\mathbf{C}$ whose entries $\bar{c}_{i,j}$ have the average number of contacts per unit time that someone in group i has with people in group j. Given:

- the population sizes of each group, N_1, N_2, and N_3
- the average number of contacts people in each group have, $\bar{c}_1$, $\bar{c}_2$, and $\bar{c}_3$
- a value of the homophily parameter $\beta \geq 1$

We can calculate the matrix $\mathbf{C}$ as follows. Starting with the first group, we set

$$q_{11} = m_1^{1/\beta}.$$

Note that when the homophily parameter $\beta = 1$, then $q_{11} = m_1$; that is, there is no tendency for members of group 1 to have more contact with others in the same group. People in group 1 have contact with others in group 1 in proportion to the share of total contacts that group a represents. Thus, when $\beta = 1$, contacts happen at random, paying no attention to group membership. When $\beta > 1$, then $q_{11} > m_1$ and people in group 1 are more likely to have contact with others in group 1 than would be expected at random.

medRxiv preprint doi: https://doi.org/10.64898/2026.01.14.26344076; this version posted January 19, 2026. The copyright holder for this preprint (which was not certified by peer review) is the author/funder, who has granted medRxiv a license to display the preprint in perpetuity.
It is made available under a CC-BY 4.0 International license .

We similarly use β to set the share of contacts that happen among members of group 2 using

$$q_{22} = m_2^{1/\beta}.$$

We then calculate rates of contact between all pairs of groups making simple assumptions. First, we fill in group 1's contacts with groups 2 and 3 by assuming that they happen in proportion to each group's share of total population contacts, m_2 and m_3. Specifically, we calculate

$$q_{12} = (1 - q_{11}) \frac{m_2}{(m_2 + m_3)}$$

and

$$q_{13} = (1 - q_{11}) \frac{m_3}{(m_2 + m_3)}.$$

We now have a complete description of group 1's contacts, given by the size of group 1 (N_1), the average number of contacts people in group 1 have ($\bar{c}_1$), and the vector $\mathbf{q}_1 = (q_{11}, q_{12}, q_{13})^T$ that describes the share of 1's contacts that takes place with each group.

We will now make use of a symmetry condition that follows from the symmetry condition described above (Equation 2): for any two groups, say i and j,

$$\begin{aligned} N_i \; \bar{c}_{ij} &= N_j \; \bar{c}_{ji} \\ \iff N_i \; \bar{c}_i \; q_{ij} &= N_j \; \bar{c}_j \; q_{ji} \\ \iff q_{ij} &= q_{ji} \; \frac{\bar{c}_j}{\bar{c}_i} \frac{N_j}{N_i}. \end{aligned}$$

Using this symmetry condition, we can deduce the rate of contacts between group 2 and group 1:

$$q_{21} = q_{12} \frac{\bar{c}_1}{\bar{c}_2} \frac{N_1}{N_2}.$$

We have expressions for q_{22} and q_{21}, so this means that $q_{23} = 1 - q_{22} - q_{21}$, and we now know everything about contacts for group 2.

Turning to group 3, we again use the symmetry condition to deduce the rate of contacts between group 3 and group 1:

$$q_{31} = q_{13} \frac{\bar{c}_1}{\bar{c}_3} \frac{N_1}{N_3}.$$

And again to deduce the rate of contacts between group 3 and group 2:

$$q_{32} = q_{23} \frac{\bar{c}_2}{\bar{c}_3} \frac{N_2}{N_3}.$$

Then we deduce $q_{33} = 1 - q_{31} - q_{32}$.

Finally, we can calculate the entries of the contact matrix $\mathbf{C}$, which has the average number of daily contacts between groups, using $\bar{c}_{ij} = \bar{c}_i \, q_{ij}$

medRxiv preprint doi: https://doi.org/10.64898/2026.01.14.26344076; this version posted January 19, 2026. The copyright holder for this preprint (which was not certified by peer review) is the author/funder, who has granted medRxiv a license to display the preprint in perpetuity.
It is made available under a CC-BY 4.0 International license .

Force of Infection

The force of infection for group i, λ_i, governs the rate at which susceptibles in group i become infected. λ_i depends on average daily contact rates that individuals in group i have with individuals in all groups, including i, and the infection prevalence for each group:

$$\lambda_i = \tau \sum_j \bar{c}_{ij} \left(\frac{\mathrm{IU}_j + \kappa\, \mathrm{IP}_j}{N_j} \right), \tag{4}$$

where κ is a parameter capturing the efficacy of protective behavior and $\bar{c}_{ij}$ is, as defined in the previous section, the average number of contacts that a single member of group i has with people in group j.

In Equation 4, we assume that (i) the $\bar{c}_{ij}$ contacts happen with unprotected and protected members of group j in proportion to the population composition of group j; and (ii) that protected and unprotected members of group j have the same average number of contacts, but that contacts with protected members are less effective at transmitting the infection.

SIR Model Equations

Here, we write out the full set of ordinary differential equations that describe the rate of movement in an out of the compartments in our SIR model.

Susceptible Unprotected ($\mathbf{SU}_i$):

$$\frac{d\mathrm{SU}_i}{dt} = -\mathrm{SU}_i\lambda_i - \pi_i\mathrm{SU}_i + \phi_i\mathrm{SP}_i + \gamma\mathrm{RU}_i \tag{5}$$

Susceptible Protected ($\mathbf{SP}_i$):

$$\frac{d\mathrm{SP}_i}{dt} = -\kappa\mathrm{SP}_i\lambda_i + \pi_i\mathrm{SU}_i - \phi_i\mathrm{SP}_i + \gamma\mathrm{RP}_i \tag{6}$$

Infected Unprotected ($\mathbf{IU}_i$):

$$\frac{d\mathrm{IU}_i}{dt} = \mathrm{SU}_i\lambda_i - \pi_i\mathrm{IU}_i + \phi_i\mathrm{IP}_i - \rho\mathrm{IU}_i \tag{7}$$

Infected Protected ($\mathbf{IP}_i$):

$$\frac{d\mathrm{IP}_i}{dt} = \kappa\mathrm{SP}_i\lambda_i + \pi_i\mathrm{IU}_i - \phi_i\mathrm{IP}_i - \rho\mathrm{IP}_i \tag{8}$$

Recovered Unprotected ($\mathbf{RU}_i$):

$$\frac{d\mathrm{RU}_i}{dt} = \rho(1-\mu_i)\mathrm{IU}_i - \pi_i\mathrm{RU}_i + \phi_i\mathrm{RP}_i - \gamma\mathrm{RU}_i \tag{9}$$

Recovered Protected ($\mathbf{RP}_i$):

$$\frac{d\mathrm{RP}_i}{dt} = \rho(1-\mu_i)\mathrm{IP}_i + \pi_i\mathrm{RU}_i - \phi_i\mathrm{RP}_i - \gamma\mathrm{RP}_i \tag{10}$$

medRxiv preprint doi: https://doi.org/10.64898/2026.01.14.26344076; this version posted January 19, 2026. The copyright holder for this preprint (which was not certified by peer review) is the author/funder, who has granted medRxiv a license to display the preprint in perpetuity.
It is made available under a CC-BY 4.0 International license .

Deceased Unprotected (DU_i):

$$\frac{d\mathrm{DU}_i}{dt} = \rho\mu_i \mathrm{IU}_i \tag{11}$$

Deceased Protected (DP_i):

$$\frac{d\mathrm{DP}_i}{dt} = \rho\mu_i \mathrm{IP}_i \tag{12}$$

0.5 Age-adjusted infection fatality rates

In our main simulations, we assume a uniform infection fatality rate (μ) across all partisan groups. However, Republicans in the BICS sample are older on average than Democrats and Independents (Table S3), and many respiratory infectious diseases exhibit strong age-mortality gradients. To examine how age composition differences might affect partisan mortality disparities, we conducted a sensitivity analysis using age-specific infection fatality rates.

We calculated group-specific infection fatality rates using the meta-regression formula from Levin et al. (2020), which estimated age-specific COVID-19 infection fatality rates:

$$\log_{10}(\mathrm{IFR}) = -3.27 + 0.0524 \times \mathrm{age} \tag{13}$$

Using the mean age of each partisan group in the BICS data, we derived the following group-specific infection fatality rates: $\mu_R = 0.0150$ for Republicans, $\mu_I = 0.0119$ for Independents, and $\mu_D = 0.0099$ for Democrats. These rates reflect the older average age of Republicans relative to Democrats in our sample.

In the SIR model, the infection fatality rate μ_i determines the proportion of infected individuals in group i who die rather than recover. Specifically, infected individuals leave the infected compartment at rate ρ, with a fraction μ_i dying and a fraction $(1-\mu_i)$ recovering:

$$\frac{d\mathrm{DU}_i}{dt} = \rho\mu_i \mathrm{IU}_i, \quad \frac{d\mathrm{DP}_i}{dt} = \rho\mu_i \mathrm{IP}_i \tag{14}$$

When we incorporate these age-adjusted fatality rates into our simulations (Figure S6), the mortality disparity between Republicans and Democrats increases dramatically—from approximately 16% higher Republican mortality (with uniform μ) to over 75% higher mortality. This amplification occurs because Republicans face a "double burden": their higher contact rates and lower adoption of protective behaviors lead to higher infection rates, and their older age distribution means a larger fraction of those infections prove fatal.

medRxiv preprint doi: https://doi.org/10.64898/2026.01.14.26344076; this version posted January 19, 2026. The copyright holder for this preprint (which was not certified by peer review) is the author/funder, who has granted medRxiv a license to display the preprint in perpetuity.
It is made available under a CC-BY 4.0 International license .

Supporting Tables

medRxiv preprint doi: https://doi.org/10.64898/2026.01.14.26344076; this version posted January 19, 2026. The copyright holder for this preprint (which was not certified by peer review) is the author/funder, who has granted medRxiv a license to display the preprint in perpetuity.
It is made available under a CC-BY 4.0 International license .

Party	% Vaccinated	Not Vaccinated		N
		% Will Get Vaccine	% Won't Get Vaccine	
Republican	57	26	73	1411
Independent	61	31	69	1295
Democrat	74	52	48	2288

Table S1: Vaccination Behavior by Political Affiliation (Wave 6). Self-reported COVID-19 vaccination status and intentions from BICS Wave 6 (May 2021), weighted for national representativeness. Among unvaccinated respondents, the table shows the percentage who reported they would or would not get the vaccine when available. Republicans showed lower vaccination rates (57%) compared to Democrats (74%), and among unvaccinated respondents, a substantially higher percentage of Republicans (73%) reported they would not get the vaccine compared to Democrats (48%).

medRxiv preprint doi: https://doi.org/10.64898/2026.01.14.26344076; this version posted January 19, 2026. The copyright holder for this preprint (which was not certified by peer review) is the author/funder, who has granted medRxiv a license to display the preprint in perpetuity.
It is made available under a CC-BY 4.0 International license .

Party	Behaviors	Apr, '20	Jun, '20	Sept, '20'	Dec, '20'	Feb, '21'	May, '21'
Democrat	Total Contacts	3.50	4.92	5.19	5.23	5.64	6.14
	Non-Household Contacts	1.79	3.10	3.47	3.37	3.72	4.28
	% Contacts with a Mask	59.60	59.71	69.70	72.71	72.30	59.85
	% Vaccinated						74.00
Independent	Total Contacts	4.12	4.93	6.04	5.47	4.84	7.23
	Non-Household Contacts	2.26	2.95	4.14	3.77	3.17	5.35
	% Contacts with a Mask	50.54	44.48	66.56	61.47	64.46	46.67
	% Vaccinated						61.00
Republican	Total Contacts	4.40	5.67	8.64	5.78	5.95	7.71
	Non-Household Contacts	2.36	3.82	6.62	3.89	4.09	5.90
	% Contacts with a Mask	47.33	51.81	64.70	61.42	57.63	45.14
	% Vaccinated						57.00
Total	Total Contacts	3.92	5.17	6.59	5.45	5.54	6.91
	Non-Household Contacts	2.07	3.30	4.72	3.62	3.70	5.06
	% Contacts with a Mask	53.38	53.03	67.05	66.56	66.01	51.85
	% Vaccinated						65.00

Table S2: Reported Pandemic Behaviors by Political Affiliation Across Waves. Unadjusted weighted averages of pandemic health behaviors by political affiliation across six BICS survey waves (April 2020 – May 2021). Total contacts and non-household contacts are reported as previous-day averages; mask use is the percentage of contacts during which respondents wore a mask; vaccination rates are from Wave 6 only (May 2021). These estimates are weighted for national representativeness but are not adjusted for demographic or geographic covariates. Republicans consistently reported more daily contacts and lower mask usage than Democrats across all waves, with the largest contact gap occurring in September 2020 (8.64 vs. 5.19 total contacts). Contact rates for all groups generally increased over time, while mask usage peaked in late 2020 before declining in 2021.

medRxiv preprint doi: https://doi.org/10.64898/2026.01.14.26344076; this version posted January 19, 2026. The copyright holder for this preprint (which was not certified by peer review) is the author/funder, who has granted medRxiv a license to display the preprint in perpetuity.
It is made available under a CC-BY 4.0 International license .

Variable	Democrats	Independents	Republicans
Average Age	46.1	46.4	50.2
Average Household Size	2.9	2.9	2.9
% Male	46.6	49.7	51.2
% White	69.9	78.1	89.8
% Hispanic	20.2	13.7	11.8
% Living in Metro	87.4	85.4	82.0
% College Degree	37.2	29.2	31.4
% Employed	59.8	54.5	58.1

Table S3: Demographic Characteristics by Political Affiliation. Unadjusted weighted averages of demographic characteristics by political affiliation from pooled BICS survey data (April 2020 – May 2021). All measures are self-reported; college degree refers to a four-year degree. Republicans in the sample were older on average (50.2 years vs. 46.1 for Democrats), more likely to be male (51.2% vs. 46.6%), more likely to be White (89.8% vs. 69.9%), and less likely to live in metropolitan areas (82.0% vs. 87.4%). These compositional differences are accounted for in the regression analyses presented in the main text.

medRxiv preprint doi: https://doi.org/10.64898/2026.01.14.26344076; this version posted January 19, 2026. The copyright holder for this preprint (which was not certified by peer review) is the author/funder, who has granted medRxiv a license to display the preprint in perpetuity.
It is made available under a CC-BY 4.0 International license .

	Dependent variable:		
	Total Contacts		
	Model 1	Model 2	Model 3
	(1)	(2)	(3)
Independent Party (ref: Democrat)	0.432**	0.452**	0.362*
	(0.192)	(0.194)	(0.202)
Republican Party (ref: Democrat)	1.364***	1.247***	1.125***
	(0.178)	(0.184)	(0.192)
Prefer Not to Answer (ref: Democrat)	1.522***	1.549***	1.800***
	(0.309)	(0.328)	(0.338)
Wave 2 (ref: Wave 1)	1.098***	1.074***	0.890**
	(0.313)	(0.360)	(0.372)
Wave 3 (ref: Wave 1)	2.427***	2.408***	2.150***
	(0.292)	(0.344)	(0.359)
Wave 4 (ref: Wave 1)	1.586***	1.677***	1.532***
	(0.295)	(0.345)	(0.356)
Wave 5 (ref: Wave 1)	1.723***	1.692***	1.724***
	(0.296)	(0.347)	(0.365)
Wave 6 (ref: Wave 1)	3.047***	2.925***	2.843***
	(0.269)	(0.325)	(0.338)
Weekend Interview	−0.227	−0.157	−0.169
	(0.149)	(0.150)	(0.155)
Age Group: [30,45) (ref: [18,30)		0.015	−0.181
		(0.221)	(0.229)
Age Group: [45,55) (ref: [18,30)		0.471*	0.386
		(0.251)	(0.261)
Age Group: [55,65) (ref: [18,30)		−0.584**	−0.589**
		(0.263)	(0.271)
Age Group: [65,100] (ref: [18,30)		−1.246***	−1.256***
		(0.259)	(0.271)
Race: Black (ref: White)		−0.754***	−0.706***
		(0.242)	(0.255)
Race: Asian (ref: White)		−1.823***	−1.700***
		(0.354)	(0.363)
Race: Other/Mixed (ref: White)		−0.711**	−0.598*
		(0.338)	(0.349)
Hispanic		0.116	−0.014
		(0.160)	(0.169)
Male		0.191	0.236
		(0.152)	(0.158)
High School Graduate (ref: College Grad)		0.217	0.128
		(0.171)	(0.178)
Less than High School (ref: College Grad)		−0.014	−0.779*
		(0.424)	(0.447)
Employed		2.580***	2.612***
		(0.171)	(0.178)
Household Size		0.819***	0.794***
		(0.047)	(0.048)
Metro Area			−0.558**
			(0.268)
CD Percent Democrat			−0.081
			(0.532)
Log Previous Week Incidence Rate			−0.147
			(0.126)
Less Strict Mask Mandate (ref: Strict Mandate)			0.939***
			(0.338)
Unspecified Mask Mandate (ref: Strict Mandate)			0.496
			(0.539)
No Mask Mandate (ref: Strict Mandate)			−0.127
			(0.401)
Constant	3.574***	0.050	0.416
	(0.274)	(0.454)	(0.708)
Observations	18,637	17,946	16,677
R^2	0.013	0.062	0.072
Adjusted R^2	0.012	0.061	0.067
Residual Std. Error	9.982 (df = 18621)	9.859 (df = 17917)	9.776 (df = 16593)
F Statistic	16.695*** (df = 15; 18621)	42.531*** (df = 28; 17917)	15.412*** (df = 83; 16593)
Note:			*p<0.1; **p<0.05; ***p<0.01

Table S4: Association between Political Party and the Number of Reported Contacts. Linear regression models predicting total daily contacts from BICS survey data (April 2020 – May 2021). Model 1 includes party affiliation and survey wave controls. Model 2 adds demographic covariates (age, race, ethnicity, gender, education, employment, household size). Model 3 adds geographic and policy controls (metropolitan area, congressional district percent Democrat, COVID-19 incidence rate, mask mandate status, and state fixed effects). Republicans reported significantly more contacts than Democrats across all model specifications, with an adjusted difference of approximately 1.1 additional daily contacts in the fully controlled model (Model 3). Standard errors in parentheses.

medRxiv preprint doi: https://doi.org/10.64898/2026.01.14.26344076; this version posted January 19, 2026. The copyright holder for this preprint (which was not certified by peer review) is the author/funder, who has granted medRxiv a license to display the preprint in perpetuity.
It is made available under a CC-BY 4.0 International license .

	Dependent variable:		
	Percentage of Contacts with a Mask		
	(1)	(2)	(3)
Independent Party (ref: Democrat)	−10.251***	−9.019***	−7.708***
	(1.043)	(1.053)	(1.090)
Republican Party (ref: Democrat)	−10.568***	−9.444***	−7.481***
	(0.938)	(0.968)	(1.003)
Prefer Not to Answer (ref: Democrat)	−14.216***	−11.109***	−10.138***
	(1.716)	(1.806)	(1.854)
Wave 2 (ref: Wave 1)	0.701	1.493	1.465
	(1.795)	(2.075)	(2.131)
Wave 3 (ref: Wave 1)	14.430***	15.161***	14.248***
	(1.688)	(1.984)	(2.064)
Wave 4 (ref: Wave 1)	12.334***	13.157***	12.987***
	(1.718)	(2.003)	(2.058)
Wave 5 (ref: Wave 1)	12.286***	13.029***	12.280***
	(1.724)	(2.016)	(2.113)
Wave 6 (ref: Wave 1)	−0.495	0.345	−0.692
	(1.561)	(1.885)	(1.947)
Weekend Interview	0.769	1.102	1.047
	(0.796)	(0.800)	(0.825)
Age Group: [30,45) (ref: [18,30)		6.421***	6.944***
		(1.167)	(1.202)
Age Group: [45,55) (ref: [18,30)		8.549***	9.616***
		(1.328)	(1.373)
Age Group: [55,65) (ref: [18,30)		3.450**	4.702***
		(1.420)	(1.462)
Age Group: [65,100] (ref: [18,30)		6.152***	6.884***
		(1.424)	(1.482)
Race: Black (ref: White)		7.258***	5.288***
		(1.302)	(1.356)
Race: Asian (ref: White)		8.479***	6.874***
		(2.085)	(2.126)
Race: Other/Mixed (ref: White)		0.998	0.816
		(1.847)	(1.913)
Hispanic		−1.175	0.657
		(0.834)	(0.882)
Male		4.268***	3.764***
		(0.810)	(0.843)
High School Graduate (ref: College Grad)		−6.206***	−4.195***
		(0.893)	(0.934)
Less than High School (ref: College Grad)		−12.987***	−9.586***
		(2.284)	(2.404)
Employed		3.592***	3.533***
		(0.941)	(0.976)
Household Size		0.632***	0.664***
		(0.242)	(0.246)
Metro Area			4.426***
			(1.400)
CD Percent Democrat			16.190***
			(2.809)
Log Previous Week Incidence Rate			0.047
			(0.678)
Less Strict Mask Mandate (ref: Strict Mandate)			2.073
			(1.792)
Unspecified Mask Mandate (ref: Strict Mandate)			−0.986
			(2.877)
No Mask Mandate (ref: Strict Mandate)			−7.073***
			(2.105)
Constant	56.435***	47.575***	37.526***
	(1.615)	(2.543)	(3.820)
Observations	13,129	12,769	11,850
R^2	0.046	0.066	0.087
Adjusted R^2	0.045	0.064	0.080
Residual Std. Error	44.147 (df = 13113)	43.644 (df = 12740)	42.975 (df = 11766)
F Statistic	42.447*** (df = 15; 13113)	32.168*** (df = 28; 12740)	13.492*** (df = 83; 11766)
Note:			*p<0.1; **p<0.05; ***p<0.01

Table S5: Association between Political Party and Reported Percentage of Contacts with a Mask. Linear regression models predicting the percentage of contacts during which respondents wore a mask, from BICS survey data (April 2020 – May 2021). Model 1 includes party affiliation and survey wave controls. Model 2 adds demographic covariates (age, race, ethnicity, gender, education, employment, household size). Model 3 adds geographic and policy controls (metropolitan area, congressional district percent Democrat, COVID-19 incidence rate, mask mandate status, and state fixed effects). Republicans reported significantly lower mask usage than Democrats across all model specifications, with an adjusted difference of approximately 7.5 percentage points lower mask use in the fully controlled model (Model 3). Standard errors in parentheses.

medRxiv preprint doi: https://doi.org/10.64898/2026.01.14.26344076; this version posted January 19, 2026. The copyright holder for this preprint (which was not certified by peer review) is the author/funder, who has granted medRxiv a license to display the preprint in perpetuity.
It is made available under a CC-BY 4.0 International license .

	Dependent variable:		
	Probability of Vaccination		
	(1)	(2)	(3)
Independent Party (ref: Democrat)	−0.138***	−0.125***	−0.123***
	(0.024)	(0.023)	(0.024)
Republican Party (ref: Democrat)	−0.152***	−0.169***	−0.161***
	(0.022)	(0.022)	(0.023)
Prefer Not to Answer (ref: Democrat)	−0.239***	−0.166***	−0.164***
	(0.035)	(0.034)	(0.035)
Weekend Interview	−0.054***	−0.043**	−0.044**
	(0.018)	(0.017)	(0.018)
Age Group: [30,45) (ref: [18,30)		−0.030	−0.028
		(0.026)	(0.027)
Age Group: [45,55) (ref: [18,30)		0.029	0.016
		(0.030)	(0.032)
Age Group: [55,65) (ref: [18,30)		0.164***	0.142***
		(0.030)	(0.031)
Age Group: [65,100] (ref: [18,30)		0.284***	0.260***
		(0.029)	(0.031)
Race: Black (ref: White)		−0.096***	−0.105***
		(0.029)	(0.031)
Race: Asian (ref: White)		0.060	0.061
		(0.045)	(0.047)
Race: Other/Mixed (ref: White)		−0.058	−0.050
		(0.040)	(0.042)
Hispanic		−0.078***	−0.057***
		(0.018)	(0.020)
Male		0.071***	0.073***
		(0.017)	(0.018)
High School Graduate (ref: College Grad)		−0.181***	−0.166***
		(0.019)	(0.020)
Less than High School (ref: College Grad)		−0.279***	−0.235***
		(0.058)	(0.063)
Employed		0.073***	0.065***
		(0.020)	(0.021)
Household Size		−0.015***	−0.014***
		(0.004)	(0.004)
Metro Area			0.012
			(0.031)
CD Percent Democrat			0.004
			(0.063)
Log Previous Week Mortality Rate			−0.042**
			(0.021)
Less Strict Mask Mandate (ref: Strict Mandate)			−0.073*
			(0.041)
Unspecified Mask Mandate (ref: Strict Mandate)			−0.154**
			(0.069)
No Mask Mandate (ref: Strict Mandate)			−0.104**
			(0.048)
Constant	0.724***	0.767***	0.826***
	(0.020)	(0.037)	(0.073)
Observations	2,680	2,680	2,497
R^2	0.049	0.168	0.192
Adjusted R^2	0.045	0.161	0.167
Residual Std. Error	0.462 (df = 2669)	0.433 (df = 2656)	0.429 (df = 2420)
F Statistic	13.647*** (df = 10; 2669)	23.302*** (df = 23; 2656)	7.561*** (df = 76; 2420)
Note:			*p<0.1; **p<0.05; ***p<0.01

Table S6: Association between Political Party and Reported Vaccination. Linear regression models predicting self-reported COVID-19 vaccination status from BICS Wave 6 data (May 2021). Vaccination data were only collected in Wave 6, when vaccines had become accessible to the general public. Model 1 includes party affiliation and survey day controls. Model 2 adds demographic covariates (age, race, ethnicity, gender, education, employment, household size). Model 3 adds geographic and policy controls (metropolitan area, congressional district percent Democrat, COVID-19 mortality rate, mask mandate status, and state fixed effects). Republicans were significantly less likely to be vaccinated than Democrats across all model specifications, with an adjusted difference of approximately 16 percentage points lower vaccination probability in the fully controlled model (Model 3). Standard errors in parentheses.

Parameter	Description	Republican	Democrat	Independent	Source
N_0	Initial population size		10,000,000		Assumed
frac_i	Fraction of total population	0.333	0.333	0.333	Assumed
β	Homophily parameter		1.000		Assumed
$\bar{c}_i$	Average daily contacts	6.750	5.340	5.780	BICS data
m_i	Mask usage (percent of contacts)	54.8	65.6	53.8	BICS data
π_i	Background rate of adopting protective behaviors	0.050	0.100	0.075	Chosen to match BICS data
ϕ_i	Rate of waning protective behaviors	0.067	0.033	0.050	Chosen to match BICS data
μ_i	Probability of death following infection	0.015	0.010	0.012	Levin et al. (2020)
γ	Rate of waning immunity		0.003		Diani et al. (2022)
ρ	Rate of leaving infected compartment (1/infectious period)		0.100		Frediani et al. (2024)
κ	Efficacy of protective behavior		0.700		Howard et al. (2021)
τ	Transmission probability per contact		0.050		Assumed

Table S7: Partisan Model Parameters. Parameter values used in the three-party SIR epidemiological model. Partisan-specific contact rates ($\bar{c}_i$) and mask usage (m_i) are derived from pooled BICS survey data (April 2020 – May 2021). Rates of adopting (π_i) and waning (ϕ_i) protective behaviors were chosen to match temporal patterns in mask usage observed in the BICS data. Infection fatality rates (μ_i) are age-adjusted using estimates from Levin et al. (2020), which found that infection fatality rates increase exponentially with age. Epidemiological parameters (waning immunity γ, infectious period ρ, mask efficacy κ) are drawn from published literature. Baseline simulations assume equal population shares across partisan groups and random mixing ($\beta = 1$). Because partisan mixing patterns could not be directly measured from BICS data and reliable estimates were not available in the literature, we vary the homophily parameter (β) across a range of values to explore how within-group contact preferences affect disease outcomes.

medRxiv preprint doi: https://doi.org/10.64898/2026.01.14.26344076; this version posted January 19, 2026. The copyright holder for this preprint (which was not certified by peer review) is the author/funder, who has granted medRxiv a license to display the preprint in perpetuity.
It is made available under a CC-BY 4.0 International license .

	Full Outbreak Size		Peak		Cumulative Deceased	
Behavior Differences	1 Year	2 Years	Magnitude	Days to Reach	1 Year	2 Years
Uniform Behavior						
Total	8,994	9,181	1,635	137	110	113
Republican	2,998	9,181	1,635	137	37	113
Independent	2,998	9,181	1,635	137	37	113
Democrat	2,998	9,181	1,635	137	37	113
Lowest Transmission Behavior						
Total	7,463	7,536	904	215	91	93
Republican	2,488	7,536	904	215	30	93
Independent	2,488	7,536	904	215	30	93
Democrat	2,488	7,536	904	215	30	93
Partisan Heterogeneity						
Mask Usage						
Total	9,013	9,213	1,653	136	111	113
Republican	3,077	9,440	1,706	135	46	142
Independent	3,003	9,208	1,651	136	36	109
Democrat	2,933	8,991	1,603	136	29	89
Contacts						
Total	8,972	9,183	1,648	135	111	114
Republican	3,150	9,683	1,763	134	47	145
Independent	2,962	9,092	1,627	135	35	108
Democrat	2,860	8,776	1,558	135	28	87
Mask + Contacts						
Total	9,023	9,326	1,710	129	110	114
Republican	3,237	10,067	1,881	128	40	124
Independent	2,980	9,252	1,689	130	36	114
Democrat	2,806	8,661	1,569	130	34	106

Table S8: Comparison of Key Outcomes Across Partisan Behavior Models. Results from three-party SIR simulations comparing disease outcomes under different behavioral assumptions. "Uniform Behavior" assumes population-average contact rates and mask usage with no partisan differences. "Lowest Transmission Behavior" assumes all groups adopt the most protective behaviors observed in the data (lowest contact rates, highest mask usage). "Partisan Heterogeneity" incorporates observed partisan differences in mask usage only, contact rates only, or both (Mask + Contacts). All simulations assume random mixing ($\beta = 1$) and an evenly split population (33% Republican, 33% Democrat, 33% Independent). Full outbreak size and cumulative deceased are shown at 1-year and 2-year time points. Peak magnitude and days to reach peak reflect the maximum infection rate during the simulation. Rates are presented per 10,000 people.

medRxiv preprint doi: https://doi.org/10.64898/2026.01.14.26344076; this version posted January 19, 2026. The copyright holder for this preprint (which was not certified by peer review) is the author/funder, who has granted medRxiv a license to display the preprint in perpetuity.
It is made available under a CC-BY 4.0 International license .

Behavior Differences	Full Outbreak Size<br>FOS	Peak<br>Magnitude	 Days to Reach	Cumulative Deceased<br>Deceased: 365
Uniform Behavior				
Total	9,360	1,869	121	114
Republican	3,120	1,869	121	38
Independent	3,120	1,869	121	38
Democrat	3,120	1,869	121	38
Partisan Differences				
Total	9,778	2,146	106	119
Republican	7,071	2,244	106	86
Independent	936	2,025	107	11
Democrat	1,771	1,887	108	22
Partisan Differences (Moderate Homophily)				
Total	9,806	2,133	103	119
Republican	7,239	2,340	102	88
Independent	899	1,866	107	11
Democrat	1,668	1,693	108	20
Partisan Differences (High Homophily)				
Total	9,833	2,098	102	119
Republican	7,320	2,382	100	89
Independent	892	1,790	108	11
Democrat	1,622	1,567	110	20

Table S9: Comparison of Key Outcomes Across Partisan Behavior Models in a Republican Majority Population. Results from three-party SIR simulations with a Republican majority population composition (70% Republican, 20% Democrat, 10% Independent). "Uniform Behavior" assumes population-average contact rates and mask usage with no partisan differences and random mixing ($\beta = 1$). "Partisan Differences" incorporates observed partisan differences in contact rates and mask usage with random mixing ($\beta = 1$). "Moderate Homophily" and "High Homophily" add increasing levels of within-group contact preference ($\beta = 3$ and $\beta = 5$, respectively). Full outbreak size (FOS) and cumulative deceased are shown at the 1-year time point. Peak magnitude and days to reach peak reflect the maximum infection rate during the simulation. Rates are presented per 10,000 people.

medRxiv preprint doi: https://doi.org/10.64898/2026.01.14.26344076; this version posted January 19, 2026. The copyright holder for this preprint (which was not certified by peer review) is the author/funder, who has granted medRxiv a license to display the preprint in perpetuity.
It is made available under a CC-BY 4.0 International license .

	Full Outbreak Size		Peak		Cumulative Deceased	
Behavior Differences	1 Year	2 Years	Magnitude	Days to Reach	1 Year	2 Years
Random Mixing						
Total	9,023	9,326	1,710	129	110	114
Republican	9,710	10,067	1,881	128	119	124
Independent	8,940	9,252	1,689	130	109	114
Democrat	8,419	8,661	1,569	130	103	106
Moderate Homophily						
Total	8,984	9,477	1,708	121	110	116
Republican	10,025	10,711	2,035	118	122	131
Independent	8,882	9,364	1,695	123	109	115
Democrat	8,046	8,356	1,478	125	98	103
High Homophily						
Total	8,986	9,636	1,667	117	110	118
Republican	10,193	11,197	2,123	111	124	137
Independent	8,883	9,484	1,689	119	109	116
Democrat	7,883	8,226	1,402	122	96	101

Table S10: Comparison of Key Outcomes Across Homophily Scenarios. Results from three-party SIR simulations with an evenly split population composition (33% Republican, 33% Democrat, 33% Independent) and observed partisan differences in contact rates and mask usage. "Random Mixing" assumes no preferential within-group contact ($\beta = 1$). "Moderate Homophily" and "High Homophily" add increasing levels of within-group contact preference ($\beta = 3$ and $\beta = 5$, respectively). Full outbreak size and cumulative deceased are shown at 1-year and 2-year time points. Peak magnitude and days to reach peak reflect the maximum infection rate during the simulation. Rates are presented per 10,000 people.

medRxiv preprint doi: https://doi.org/10.64898/2026.01.14.26344076; this version posted January 19, 2026. The copyright holder for this preprint (which was not certified by peer review) is the author/funder, who has granted medRxiv a license to display the preprint in perpetuity.
It is made available under a CC-BY 4.0 International license .

Behavior Differences	Full Outbreak Size<br>FOS	Peak<br>Magnitude	 Days to Reach	Cumulative Deceased<br>Deceased: 365
Uniform Behavior				
Total	8,710	1,463	150	107
Republican	2,903	1,463	150	36
Independent	2,903	1,463	150	36
Democrat	2,903	1,463	150	36
Partisan Differences				
Total	8,397	1,348	156	103
Republican	1,888	1,555	154	23
Independent	861	1,389	156	11
Democrat	5,648	1,286	157	69
Partisan Differences (Moderate Homophily)				
Total	8,326	1,353	141	102
Republican	1,977	1,746	136	24
Independent	893	1,553	138	11
Democrat	5,456	1,252	144	67
Partisan Differences (High Homophily)				
Total	8,307	1,311	132	102
Republican	2,023	1,878	122	25
Independent	895	1,628	127	11
Democrat	5,389	1,211	137	66

Table S11: Comparison of Key Outcomes Across Partisan Behavior Models in a Democrat Majority Population. Results from three-party SIR simulations with a Democrat majority population composition (20% Republican, 70% Democrat, 10% Independent). "Uniform Behavior" assumes population-average contact rates and mask usage with no partisan differences and random mixing ($\beta = 1$). "Partisan Differences" incorporates observed partisan differences in contact rates and mask usage with random mixing ($\beta = 1$). "Moderate Homophily" and "High Homophily" add increasing levels of within-group contact preference ($\beta = 3$ and $\beta = 5$, respectively). Full outbreak size (FOS) and cumulative deceased are shown at the 1-year time point. Peak magnitude and days to reach peak reflect the maximum infection rate during the simulation. Rates are presented per 10,000 people.

medRxiv preprint doi: https://doi.org/10.64898/2026.01.14.26344076; this version posted January 19, 2026. The copyright holder for this preprint (which was not certified by peer review) is the author/funder, who has granted medRxiv a license to display the preprint in perpetuity.
It is made available under a CC-BY 4.0 International license .

medRxiv preprint doi: https://doi.org/10.64898/2026.01.14.26344076; this version posted January 19, 2026. The copyright holder for this preprint (which was not certified by peer review) is the author/funder, who has granted medRxiv a license to display the preprint in perpetuity.
It is made available under a CC-BY 4.0 International license .

Political Party	Standard Deviation	Variance	Coefficient of Variation	Mean Contacts
Republican	1.62	2.62	0.26	6.31
Democrat	0.81	0.66	0.16	5.14
Independent	1.04	1.07	0.19	5.48
Levene Test	F = 15.471	df = 2		
p-value		0	Significant*	

Table S12: Volatility Measures and Levene's Test for Total Contacts by Political Party. Measures of variability in average daily contact rates across six BICS survey waves (April 2020 – May 2021). Republicans showed the highest volatility in contact patterns (coefficient of variation = 0.26) compared to Independents (0.19) and Democrats (0.16). Levene's test confirms that these differences in variance are statistically significant ($F = 15.47$, $df = 2$, $p < 0.001$), indicating that Republicans' contact behaviors fluctuated more over time than those of other partisan groups. *Significant at $p < 0.001$.

medRxiv preprint doi: https://doi.org/10.64898/2026.01.14.26344076; this version posted January 19, 2026. The copyright holder for this preprint (which was not certified by peer review) is the author/funder, who has granted medRxiv a license to display the preprint in perpetuity.
It is made available under a CC-BY 4.0 International license .

Supporting Figures

medRxiv preprint doi: https://doi.org/10.64898/2026.01.14.26344076; this version posted January 19, 2026. The copyright holder for this preprint (which was not certified by peer review) is the author/funder, who has granted medRxiv a license to display the preprint in perpetuity.
It is made available under a CC-BY 4.0 International license .

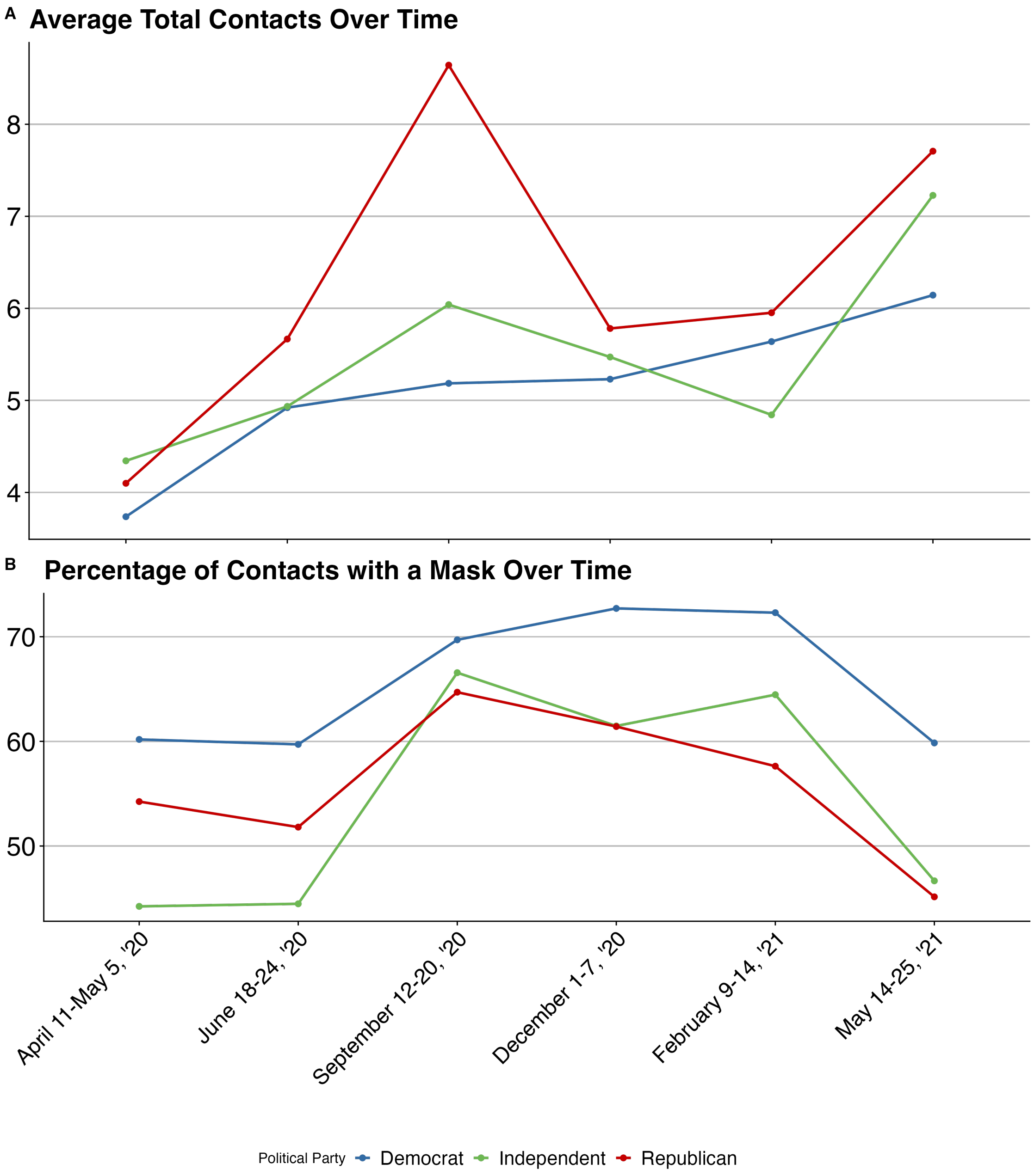

Figure S1: Partisan Health Behaviors Over Time. Panel (A) shows average daily in-person contacts by partisan affiliation across six BICS survey waves (April 2020 – May 2021). Panel (B) shows the percentage of contacts involving mask use by partisan affiliation over the same period. Republicans (red), Democrats (blue), and Independents (green) are shown with 95% confidence intervals. Contact rates and masking behaviors fluctuated over time in response to pandemic conditions, with persistent partisan differences throughout the study period.

medRxiv preprint doi: https://doi.org/10.64898/2026.01.14.26344076; this version posted January 19, 2026. The copyright holder for this preprint (which was not certified by peer review) is the author/funder, who has granted medRxiv a license to display the preprint in perpetuity.
It is made available under a CC-BY 4.0 International license .

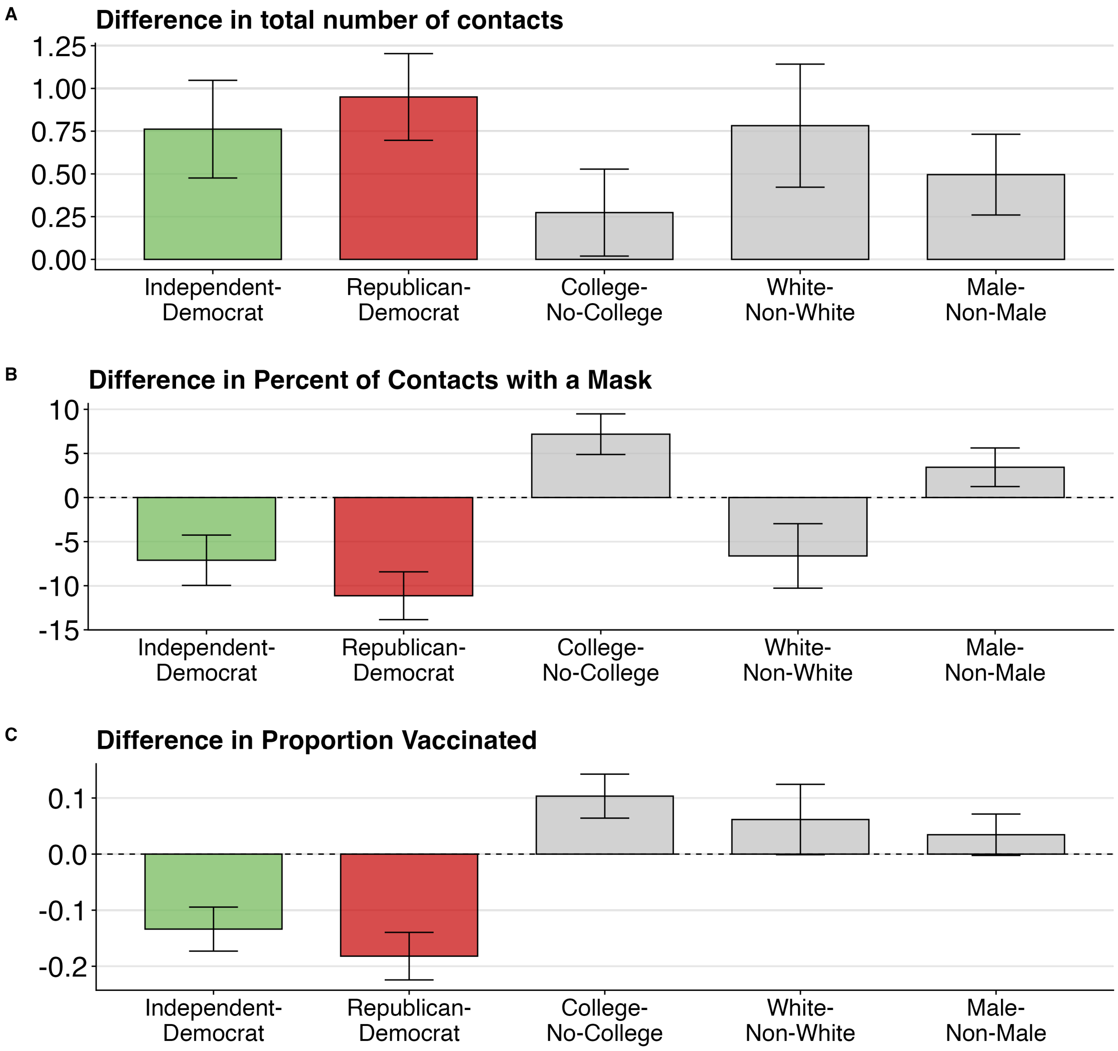

Figure S2: Partisan and Demographic Group Differences in Health Behaviors Among Adults Age 65 and Older. Comparison of partisan and demographic group differences for three health behaviors: (A) total reported in-person contacts from the previous day; (B) percentage of contacts involving mask use among those reporting previous-day contacts, based on detailed information from three selected contacts per respondent; and (C) estimated vaccination rates reported in Wave 6 of BICS in May 2021. Effect sizes are coefficients from univariate linear regression models estimated separately for each demographic characteristic and behavior. Sample includes respondents age 65 and older across six BICS survey waves (April 2020 – May 2021), except Panel C. Error bars show 95% confidence intervals.

medRxiv preprint doi: https://doi.org/10.64898/2026.01.14.26344076; this version posted January 19, 2026. The copyright holder for this preprint
(which was not certified by peer review) is the author/funder, who has granted medRxiv a license to display the preprint in perpetuity.
It is made available under a CC-BY 4.0 International license .

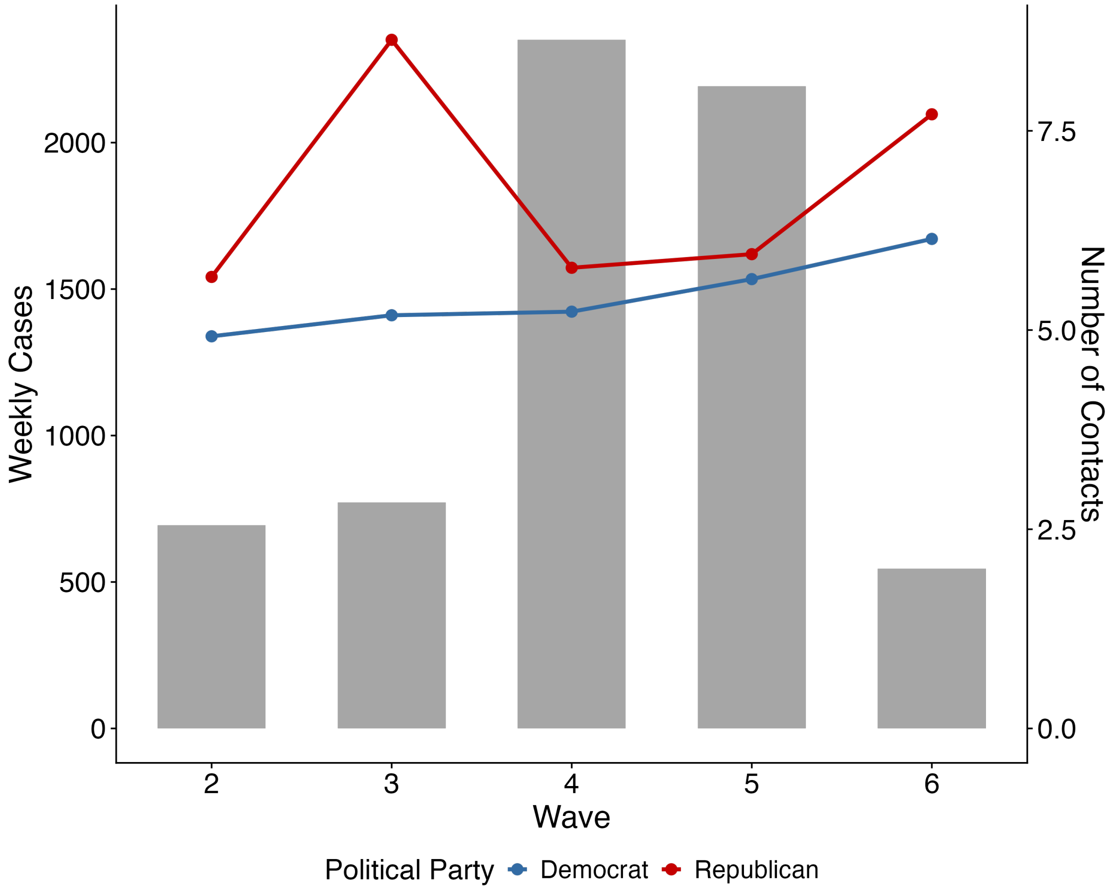

Figure S3: Partisan Contacts and National Weekly COVID-19 Cases Over Time. Average daily in-person contacts by partisan affiliation (left axis) shown alongside national weekly COVID-19 case counts (right axis, gray bars) across six BICS survey waves (April 2020 – May 2021). Republicans (red), Democrats (blue), and Independents (green) are shown with 95% confidence intervals. Contact rates for all partisan groups generally declined during periods of high case counts, though Republicans consistently maintained higher contact levels than Democrats throughout the study period.

medRxiv preprint doi: https://doi.org/10.64898/2026.01.14.26344076; this version posted January 19, 2026. The copyright holder for this preprint (which was not certified by peer review) is the author/funder, who has granted medRxiv a license to display the preprint in perpetuity.
It is made available under a CC-BY 4.0 International license .

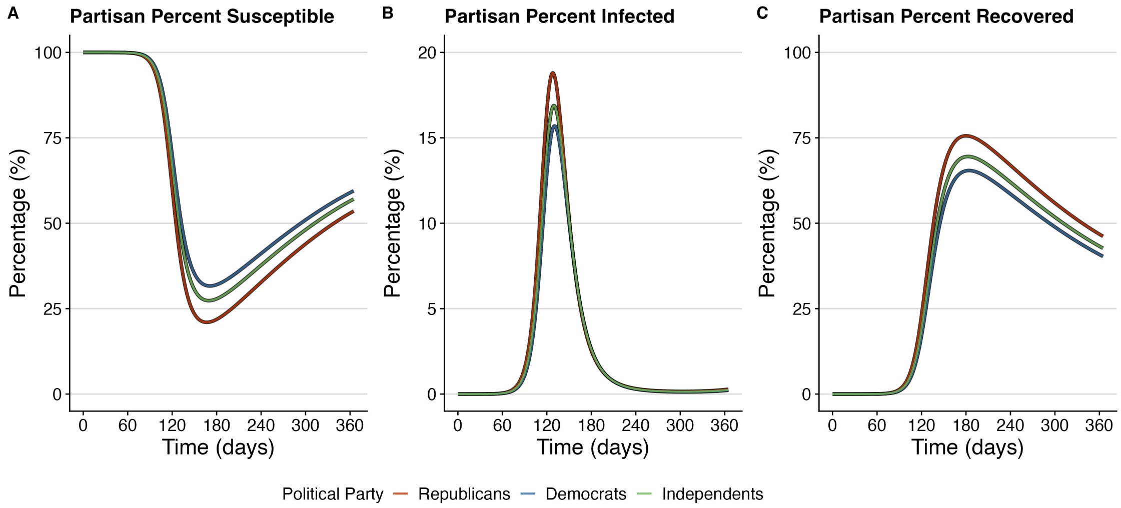

Figure S4: Multi-Panel Epidemic Outcomes Incorporating Partisan Behavioral Patterns Over Time. Results from a three-party SIR epidemiological model incorporating behavioral data pooled from all six BICS survey waves (April 2020 – May 2021), assuming random partisan mixing ($\beta = 1$) and an evenly split electorate over a one-year simulation. Panel (A) shows daily incidence rates per 10,000 for Republicans (red), Democrats (blue), and Independents (green). Panel (B) shows cumulative incidence over time. Panel (C) shows cumulative mortality. Panel (D) shows the percentage of each group currently infected over time. All model parameters sourced from published literature as detailed in Table S7.

medRxiv preprint doi: https://doi.org/10.64898/2026.01.14.26344076; this version posted January 19, 2026. The copyright holder for this preprint (which was not certified by peer review) is the author/funder, who has granted medRxiv a license to display the preprint in perpetuity.
It is made available under a CC-BY 4.0 International license .

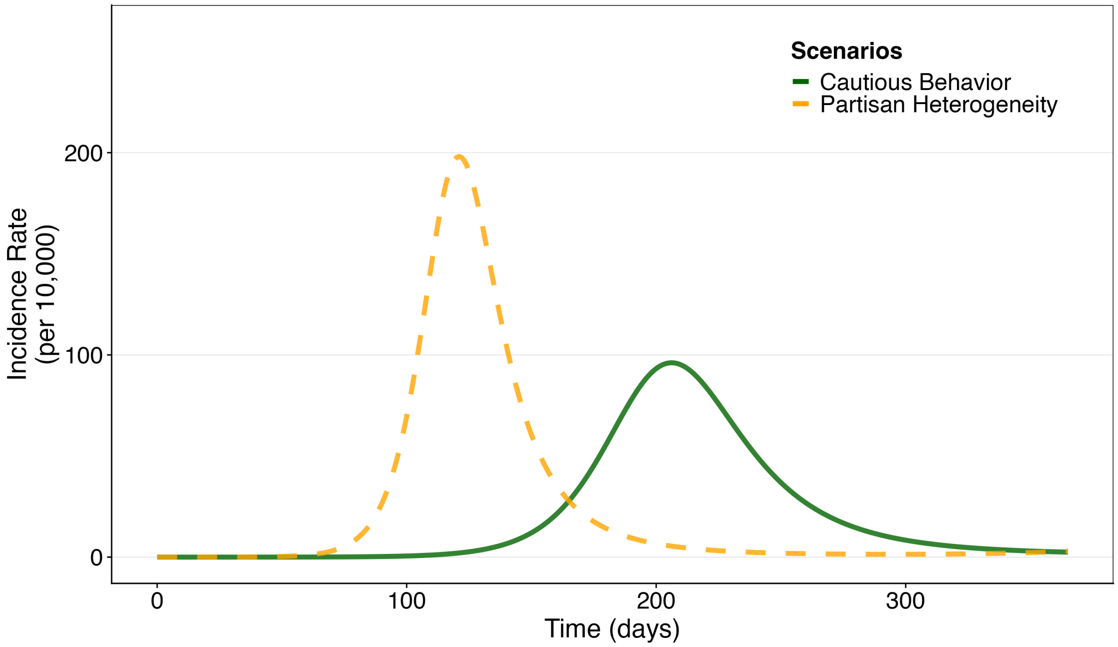

Figure S5: Sensitivity Analysis: Epidemic Outcomes Under Increased Protective Behavior Adoption. Results from a three-party SIR model with elevated background rates of protective behavior adoption (π_i) compared to baseline estimates. This scenario examines how increased caution across all partisan groups affects epidemic dynamics, including incidence rates, cumulative infections, and mortality over a one-year simulation. All other model parameters remain as specified in Table S7.

medRxiv preprint doi: https://doi.org/10.64898/2026.01.14.26344076; this version posted January 19, 2026. The copyright holder for this preprint (which was not certified by peer review) is the author/funder, who has granted medRxiv a license to display the preprint in perpetuity.
It is made available under a CC-BY 4.0 International license .

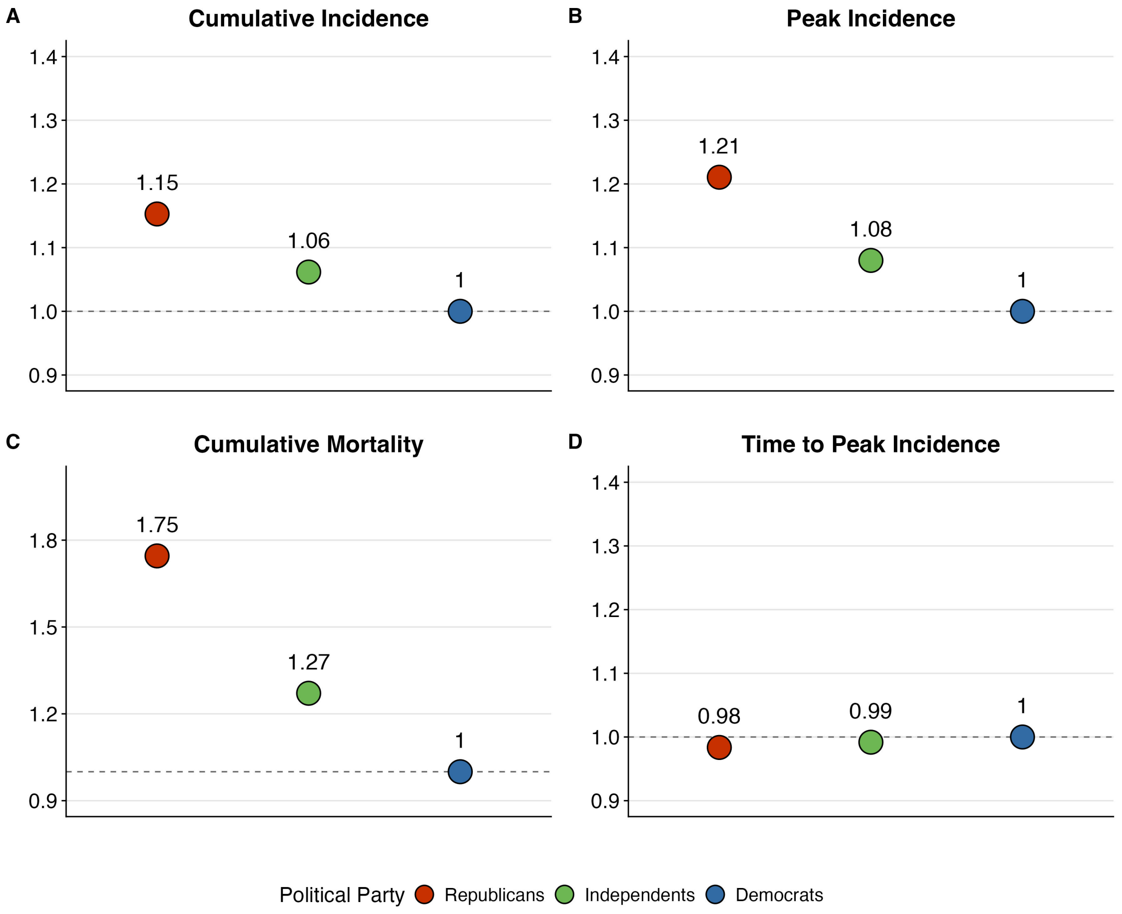

Figure S6: Results from a three-party SIR epidemiological model incorporating data pooled from all six BICS survey waves (April 2020-May 2021), assuming random partisan mixing and an evenly split electorate over a one-year simulation. The model incorporates differential mortality by assigning higher average infection mortality rates for older groups (Republicans) as outlined in the methods, reflecting that a COVID-like disease would be deadlier for older people. Data points are shown relative to the reference group (Democrats), with Democrats always equal to 1. Values greater than 1 indicate numbers larger than Democrats whereas values less than 1 indicate numbers smaller relative to Democrats. Panel (A) displays relative cumulative incidence. Panel (B) displays the relative cumulative mortality. Panel (C) displays the relative peak percentage of each group infected. Panel (D) displays the relative time to reach peak infection. All model parameters sourced from published literature as detailed in Table S7.

medRxiv preprint doi: https://doi.org/10.64898/2026.01.14.26344076; this version posted January 19, 2026. The copyright holder for this preprint (which was not certified by peer review) is the author/funder, who has granted medRxiv a license to display the preprint in perpetuity.
It is made available under a CC-BY 4.0 International license .

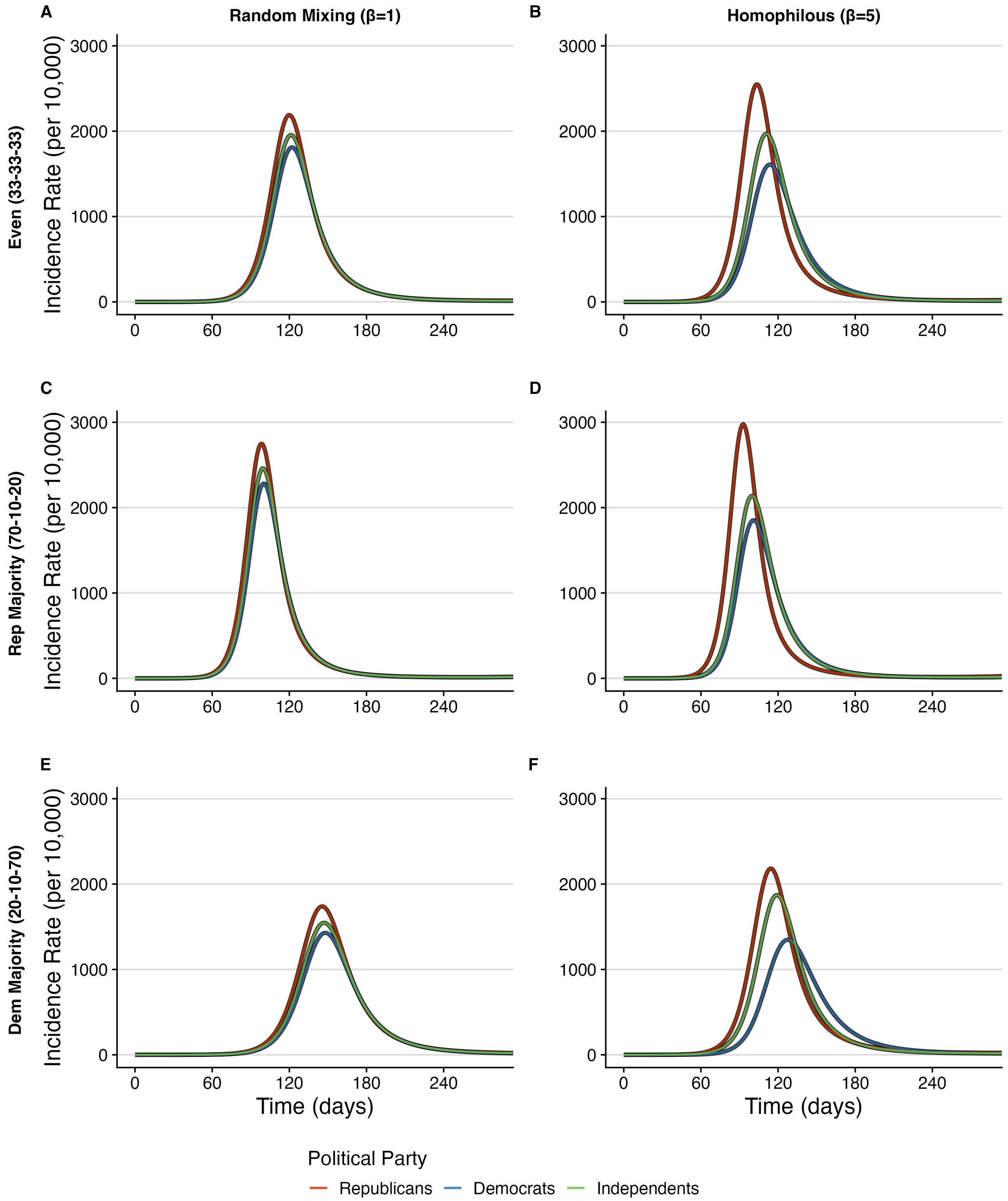

Figure S7: Partisan-specific incidence rates from SIR epidemiological models incorporating data from all six BICS survey waves (April 2020-May 2021). Left column: random mixing ($\beta = 1$); right column: homophilous mixing ($\beta = 5$). Rows show different population compositions: (A-B) even electorate (33-33-33), (C-D) Republican majority (70-10-20), (E-F) Democrat majority (20-10-70). Republican (red), Democrat (blue), and Independent (green) incidence rates per 10,000 shown over one year. Partisan disparities in infection rates increase with both Republican population share and homophily.

medRxiv preprint doi: https://doi.org/10.64898/2026.01.14.26344076; this version posted January 19, 2026. The copyright holder for this preprint (which was not certified by peer review) is the author/funder, who has granted medRxiv a license to display the preprint in perpetuity.
It is made available under a CC-BY 4.0 International license .

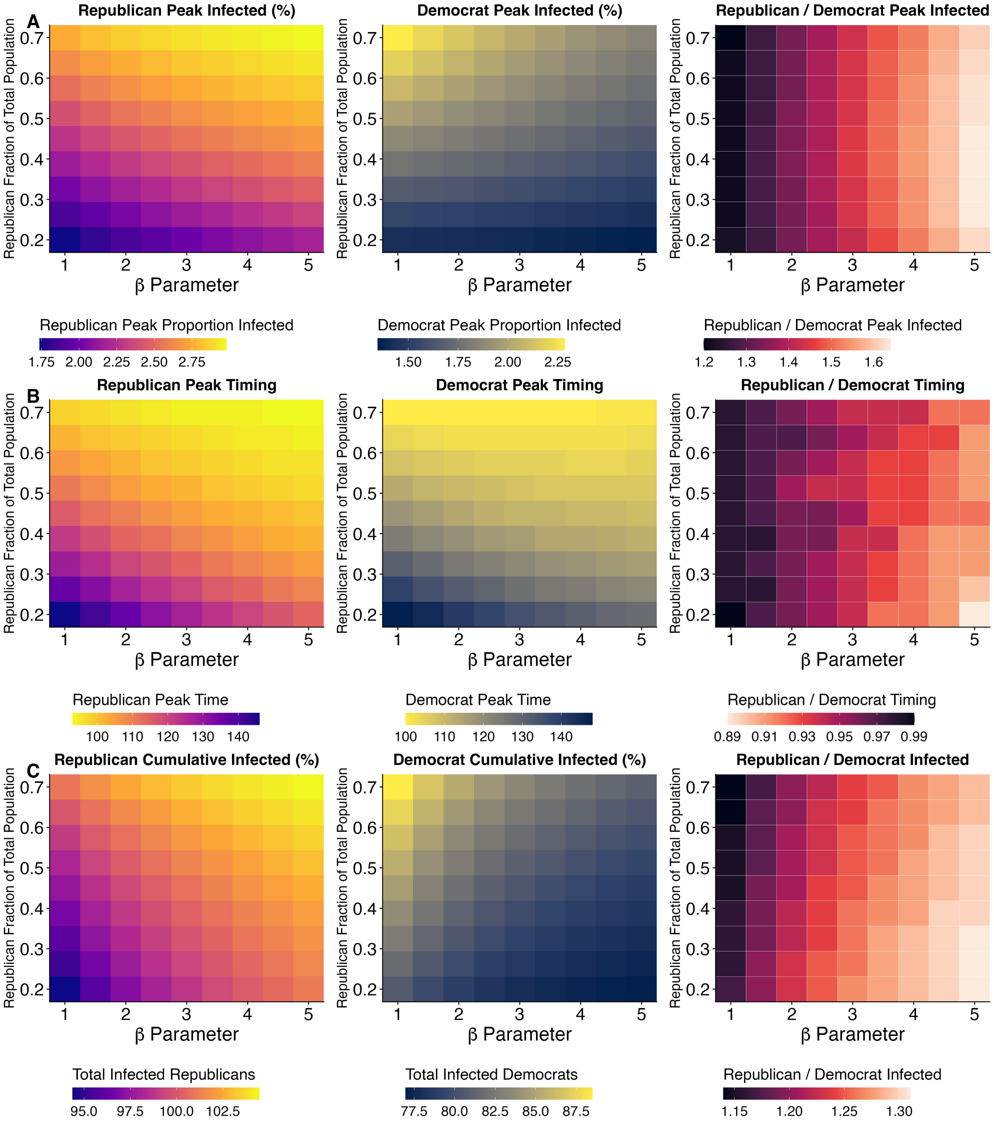

Figure S8: The effect of varying partisan homophily and population composition on peak infected. Results from sensitivity analysis varying Republican homophily parameters (β: 1.0-5.0, 51 steps) and population fractions (Republican: 20-70%, Democrat: 70-20%). Each simulation uses the three-party SIR model with base parameters from Table S7 to examine epidemic outcomes including peak infection rates, cumulative incidence, and mortality by partisan group.

medRxiv preprint doi: https://doi.org/10.64898/2026.01.14.26344076; this version posted January 19, 2026. The copyright holder for this preprint (which was not certified by peer review) is the author/funder, who has granted medRxiv a license to display the preprint in perpetuity.
It is made available under a CC-BY 4.0 International license .

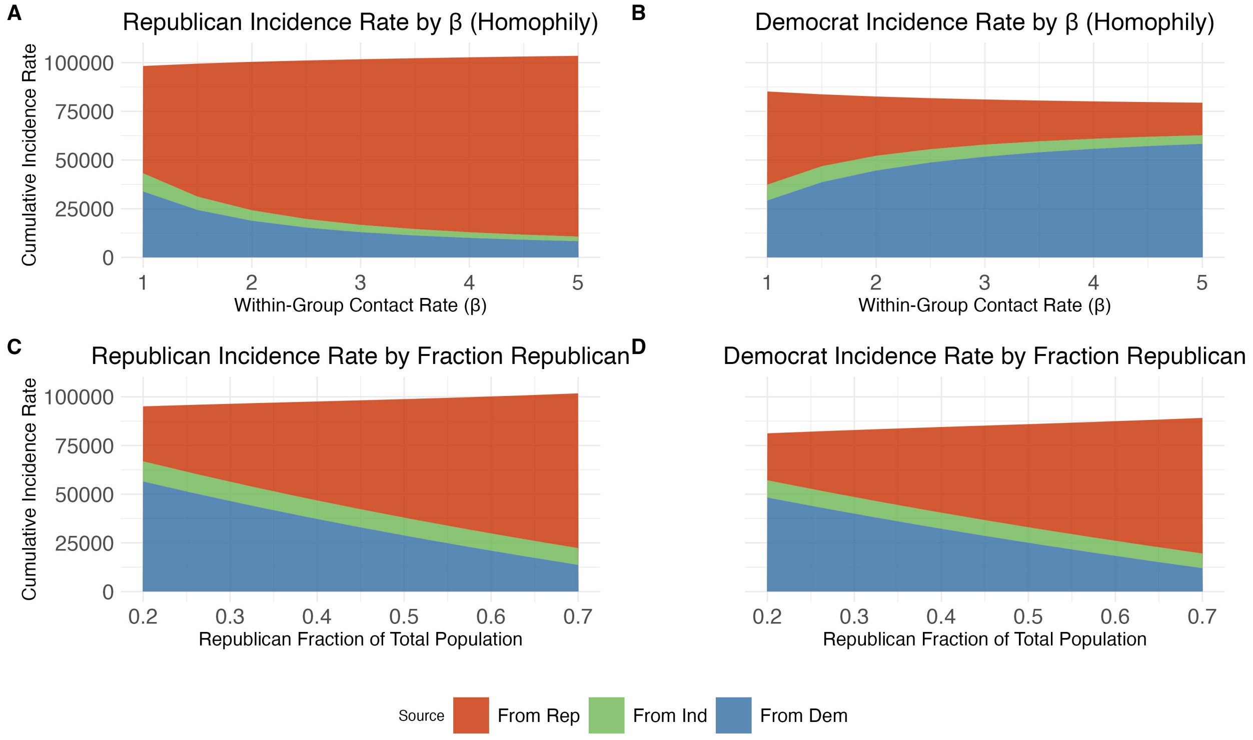

Figure S9: The effect of varying partisan homophily and population composition on outbreak size. Results from sensitivity analysis varying Republican homophily parameters (β: 1.0-5.0, 51 steps) and population fractions (Republican: 20-70%, Democrat: 70-20%). Each simulation uses the three-party SIR model with base parameters from Table S7 to examine outbreak size by partisan group. Panel (A) shows Republican outbreak size as a function of homophily (β), (B) shows Democrat outbreak size as a function of homophily (β), (C) shows Republican outbreak size as a function of Republican population fraction, and (D) shows Democrat outbreak size as a function of Republican population fraction.

medRxiv preprint doi: https://doi.org/10.64898/2026.01.14.26344076; this version posted January 19, 2026. The copyright holder for this preprint
(which was not certified by peer review) is the author/funder, who has granted medRxiv a license to display the preprint in perpetuity.
It is made available under a CC-BY 4.0 International license .

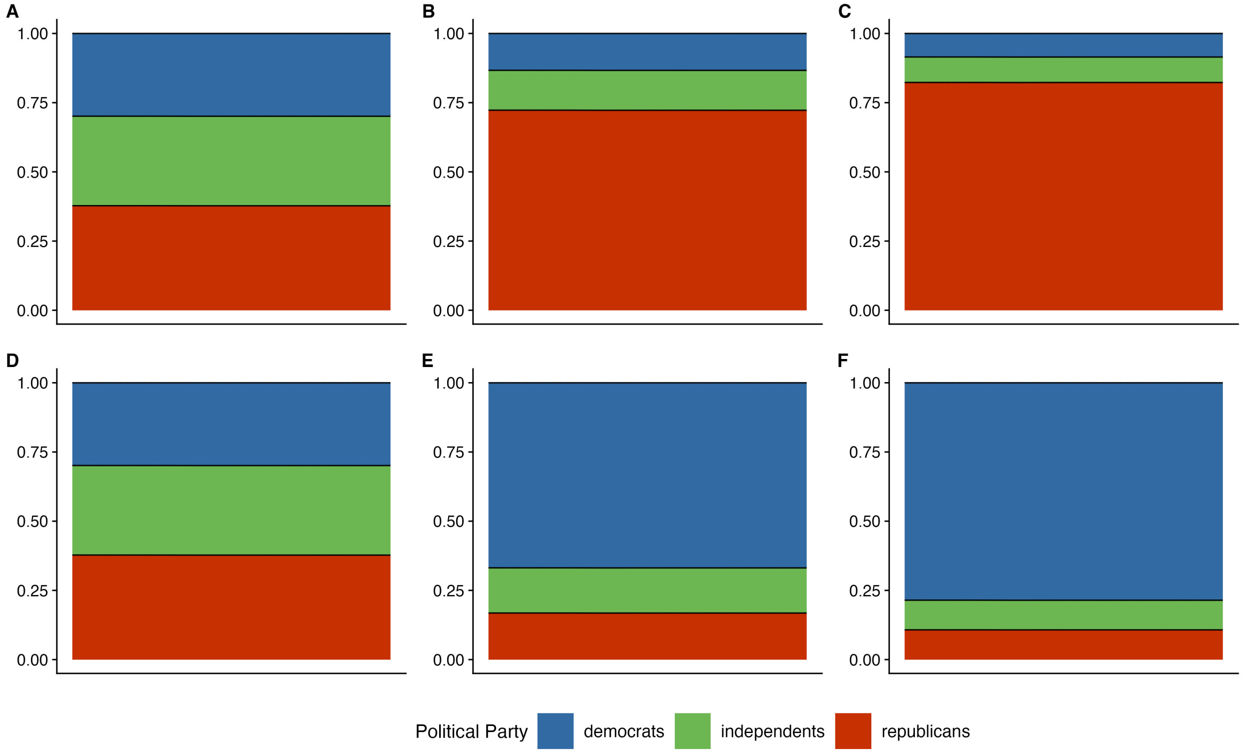

Figure S10: Share of contacts with other partisan groups based on homophily parameters (β = 1, 3, 5) in an evenly split population. Each stacked bar shows the proportion of contacts with Democrats (blue), Independents (green), and Republicans (red). Panels (A), (B), and (C) show Republican contact patterns under no homophily (β=1), moderate homophily (β=3), and high homophily (β=5), respectively. Panels (D), (E), and (F) show corresponding Democrat contact patterns under the same homophily conditions. As homophily increases, both Republicans and Democrats contact more members of their own group and fewer from opposing groups.

medRxiv preprint doi: https://doi.org/10.64898/2026.01.14.26344076; this version posted January 19, 2026. The copyright holder for this preprint (which was not certified by peer review) is the author/funder, who has granted medRxiv a license to display the preprint in perpetuity.
It is made available under a CC-BY 4.0 International license .

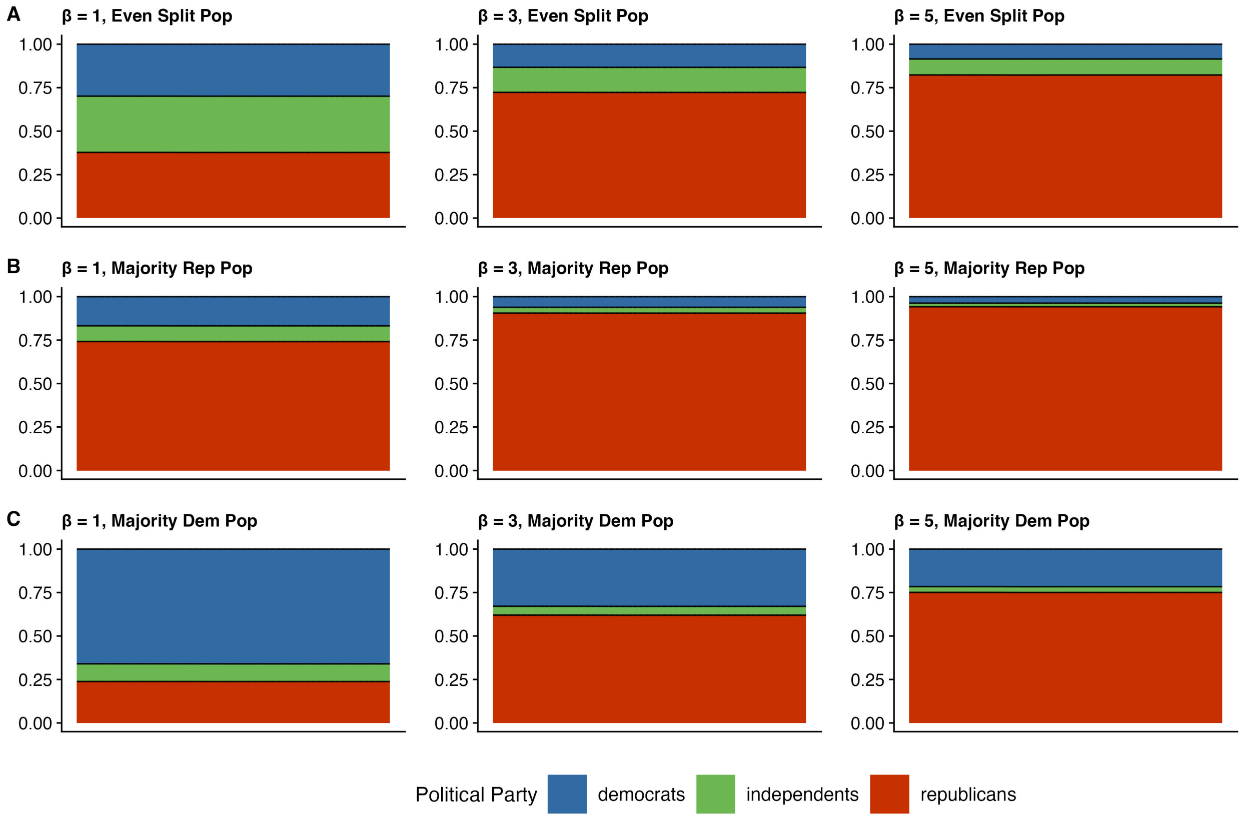

Figure S11: Republican Contact Patterns Under Varying Homophily and Population Composition. Share of Republican contacts with other partisan groups based on homophily parameters ($\beta = 1, 3, 5$). Each stacked bar shows the proportion of Republican contacts with Democrats (blue), Independents (green), and other Republicans (red). Row A displays results for an evenly split population, Row B for a Republican majority population, and Row C for a Democrat majority population. Columns show increasing homophily from left to right: no homophily (β=1), moderate homophily (β=3), and high homophily (β=5). Even without homophily, Republicans have more same-party contacts in Republican-majority populations due to baseline demographics. As homophily increases, Republicans increasingly contact members of their own group while reducing contacts with opposing partisan groups.

medRxiv preprint doi: https://doi.org/10.64898/2026.01.14.26344076; this version posted January 19, 2026. The copyright holder for this preprint (which was not certified by peer review) is the author/funder, who has granted medRxiv a license to display the preprint in perpetuity.
It is made available under a CC-BY 4.0 International license .

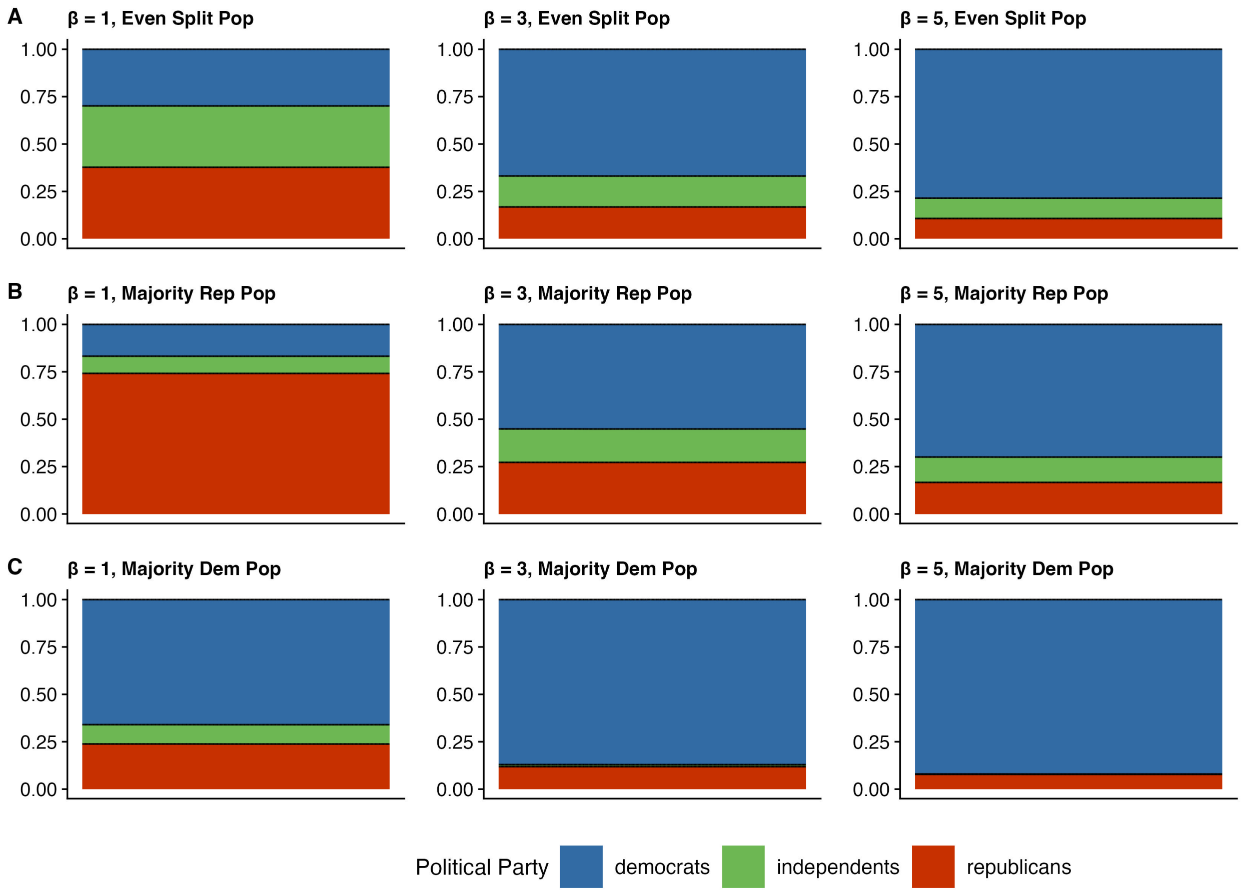

Figure S12: Democrat Contact Patterns Under Varying Homophily and Population Composition. Share of Democrat contacts with other partisan groups based on homophily parameters ($\beta = 1, 3, 5$). Each stacked bar shows the proportion of Democrat contacts with Democrats (blue), Independents (green), and Republicans (red). Row A displays results for an evenly split population, Row B for a Republican majority population, and Row C for a Democrat majority population. Columns show increasing homophily from left to right: no homophily (β=1), moderate homophily (β=3), and high homophily (β=5). Even without homophily, Democrats have more same-party contacts in Democrat-majority populations due to baseline demographics. As homophily increases, Democrats increasingly contact members of their own group while reducing contacts with opposing partisan groups.

medRxiv preprint doi: https://doi.org/10.64898/2026.01.14.26344076; this version posted January 19, 2026. The copyright holder for this preprint (which was not certified by peer review) is the author/funder, who has granted medRxiv a license to display the preprint in perpetuity.
It is made available under a CC-BY 4.0 International license .

References

Brown, Jacob R. and Ryan D. Enos. 2021. "The measurement of partisan sorting for 180 million voters." *Nature Human Behaviour* 5(8):998–1008. Publisher: Nature Publishing Group.
URL: *https://www.nature.com/articles/s41562-021-01066-z*

Currarini, Sergio, Matthew O. Jackson and Paolo Pin. 2010. "Identifying the roles of race-based choice and chance in high school friendship network formation." *Proceedings of the National Academy of Sciences* 107(11):4857–4861. Publisher: Proceedings of the National Academy of Sciences.
URL: *https://www.pnas.org/doi/abs/10.1073/pnas.0911793107*

Diani, Sara, Erika Leonardi, Attilio Cavezzi, Simona Ferrari, Oriana Iacono, Alice Limoli, Zoe Bouslenko, Daniele Natalini, Stefania Conti, Mauro Mantovani, Silvano Tramonte, Alberto Donzelli and Eugenio Serravalle. 2022. "SARS-CoV-2-The Role of Natural Immunity: A Narrative Review." *Journal of Clinical Medicine* 11(21):6272.

Frediani, Jennifer K., Richard Parsons, Kaleb B. McLendon, Adrianna L. Westbrook, Wilbur Lam, Greg Martin and Nira R. Pollock. 2024. "The New Normal: Delayed Peak SARS-CoV-2 Viral Loads Relative to Symptom Onset and Implications for COVID-19 Testing Programs." *Clinical Infectious Diseases: An Official Publication of the Infectious Diseases Society of America* 78(2):301–307.

Glass, Laura M. and Robert J. Glass. 2008. "Social contact networks for the spread of pandemic influenza in children and teenagers." *BMC Public Health* 8(1):61.
URL: *https://doi.org/10.1186/1471-2458-8-61*

Howard, Jeremy, Austin Huang, Zhiyuan Li, Zeynep Tufekci, Vladimir Zdimal, Helene-Mari van der Westhuizen, Arne von Delft, Amy Price, Lex Fridman, Lei-Han Tang, Viola Tang, Gregory L. Watson, Christina E. Bax, Reshama Shaikh, Frederik Questier, Danny Hernandez, Larry F. Chu, Christina M. Ramirez and Anne W. Rimoin. 2021. "An evidence review of face masks against COVID-19." *Proceedings of the National Academy of Sciences* 118(4):e2014564118. Publisher: Proceedings of the National Academy of Sciences.
URL: *https://www.pnas.org/doi/abs/10.1073/pnas.2014564118*

Huang, Chunlin, Xingwu Liu, Shiwei Sun, Shuai Cheng Li, Minghua Deng, Guangxue He, Haicang Zhang, Chao Wang, Yang Zhou, Yanlin Zhao and Dongbo Bu. 2016. "Insights into the transmission of respiratory infectious diseases through empirical human contact networks." *Scientific Reports* 6(1):31484. Publisher: Nature Publishing Group.
URL: *https://www.nature.com/articles/srep31484*

Levin, Andrew T., William P. Hanage, Nana Owusu-Boaitey, Kensington B. Cochran, Seamus P. Walsh and Gideon Meyerowitz-Katz. 2020. "Assessing the age specificity of infection fatality rates for COVID-19: systematic review, meta-analysis, and public policy implications." *European Journal of Epidemiology* 35(12):1123–1138. Company: Springer Distributor: Springer Institution: Springer Label: Springer Number: 12 Publisher: Springer

medRxiv preprint doi: https://doi.org/10.64898/2026.01.14.26344076; this version posted January 19, 2026. The copyright holder for this preprint (which was not certified by peer review) is the author/funder, who has granted medRxiv a license to display the preprint in perpetuity.
It is made available under a CC-BY 4.0 International license .

Netherlands.
URL: *https://link.springer.com/article/10.1007/s10654-020-00698-1*

McPherson, Miller, Lynn Smith-Lovin and James M. Cook. 2001. "Birds of a Feather: Homophily in Social Networks." *Annual Review of Sociology* 27:415–444. Publisher: Annual Reviews.
URL: *https://www.jstor.org/stable/2678628*

Mossong, Joël, Niel Hens, Mark Jit, Philippe Beutels, Kari Auranen, Rafael Mikolajczyk, Marco Massari, Stefania Salmaso, Gianpaolo Scalia Tomba, Jacco Wallinga, Janneke Heijne, Malgorzata Sadkowska-Todys, Magdalena Rosinska and W. John Edmunds. 2008. "Social Contacts and Mixing Patterns Relevant to the Spread of Infectious Diseases." *PLOS Medicine* 5(3):e74. Publisher: Public Library of Science.
URL: *https://journals.plos.org/plosmedicine/article?id=10.1371/journal.pmed.0050074*

Pasquale, Dana K., Whitney Welsh, Keisha L. Bentley-Edwards, Andrew Olson, Madelynn C. Wellons and James Moody. 2024. "Homophily and social mixing in a small community: Implications for infectious disease transmission." *PLOS ONE* 19(5):e0303677. Publisher: Public Library of Science.
URL: *https://journals.plos.org/plosone/article?id=10.1371/journal.pone.0303677*

Potter, Gail E., Timo Smieszek and Kerstin Sailer. 2015. "Modeling workplace contact networks: The effects of organizational structure, architecture, and reporting errors on epidemic predictions." *Network Science* 3(3):298–325.
URL: *https://www.cambridge.org/core/journals/network-science/article/modeling-workplace-contact-networks-the-effects-of-organizational-structure-architecture-and-reporting-errors-on-epidemic-predictions/B07ED3DFF0C2A4A18F4D58180AA08A29*
